# Eukaryote DIRS1-like retrotransposons: an overview

**DOI:** 10.1186/1471-2164-12-621

**Published:** 2011-12-20

**Authors:** Mathieu Piednoël, Isabelle R Gonçalves, Dominique Higuet, Eric Bonnivard

**Affiliations:** 1 UMR 7138 Systématique Adaptation Evolution, Equipe Génétique et Evolution, Université Pierre et Marie Curie Paris 6, Case 5, Bâtiment A, porte 427, 7 quai St Bernard, 75252 Paris Cedex 05, France

## Abstract

**Background:**

DIRS1-like elements compose one superfamily of tyrosine recombinase-encoding retrotransposons. They have been previously reported in only a few diverse eukaryote species, describing a patchy distribution, and little is known about their origin and dynamics. Recently, we have shown that these retrotransposons are common among decapods, which calls into question the distribution of DIRS1-like retrotransposons among eukaryotes.

**Results:**

To determine the distribution of DIRS1-like retrotransposons, we developed a new computational tool, ReDoSt, which allows us to identify well-conserved DIRS1-like elements. By screening 274 completely sequenced genomes, we identified more than 4000 DIRS1-like copies distributed among 30 diverse species which can be clustered into roughly 300 families. While the diversity in most species appears restricted to a low copy number, a few bursts of transposition are strongly suggested in certain species, such as *Danio rerio *and *Saccoglossus kowalevskii*.

**Conclusion:**

In this study, we report 14 new species and 8 new higher taxa that were not previously known to harbor DIRS1-like retrotransposons. Now reported in 61 species, these elements appear widely distributed among eukaryotes, even if they remain undetected in streptophytes and mammals. Especially in unikonts, a broad range of taxa from Cnidaria to Sauropsida harbors such elements. Both the distribution and the similarities between the DIRS1-like element phylogeny and conventional phylogenies of the host species suggest that DIRS1-like retrotransposons emerged early during the radiation of eukaryotes.

## Background

**Figure 1:**
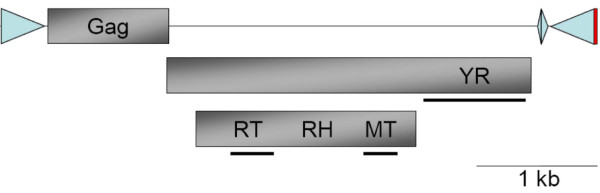
**Structure of the DIRS1 reference element identified in the slime mold *Dictyostelium discoideum***. The three ORFs encoding the GAG, tyrosine recombinase (YR) and *pol *(Reverse Transcriptase (RT) - RNase H (RH) - MethylTransferase (MT) domain series) regions correspond to shaded boxes. The two Inverted Terminal Repeats (ITRs) are represented by the outer triangles. The two Internal Complementary Regions (ICRs) correspond to the inner triangles. The rE at the 3'-end of the element is represented by the red box. The positions of the three alignment profiles used to screen for DIRS1-like elements among genomes are symbolized by black bars under their respective domains (RT-, MT- and YR-encoding domains).

The tyrosine recombinase (YR)-encoding elements constitute one of the major groups of retrotransposons [1,2]. These elements encode a YR that is required for the mechanism of integration into the genome [3], distinguishing them from other retrotransposons (*i.e*., LTR retrotransposons, LINEs, SINEs and Penelope) [4]. DIRS1-like retrotransposons belong to the YR-encoding element superfamilies [5], whose constituents exhibit a unique structure made up of three ORFs and uncommon repeats (Figure 1). The first ORF encodes a putative GAG protein, the second the YR, and the third a *pol *region composed of three distinct domains: a reverse transcriptase (RT), a RNase H (RH), and a methyltransferase (MT). The function of this latter still remains unknown. Depending on the element considered, there may be considerable overlap between the *pol *and the YR regions (Figure 1). The catalytic tyrosine recombinase domain is encoded by the non-overlapping 3'-end of the YR ORF. Many phylogenetic relationship analyses have shown that the RT/RH domains of DIRS1-like retrotransposons are closely related to those of Ty3/Gypsy LTR retrotransposons, suggesting that all these elements diverged from an ancient GAG-*pol *form of retrotransposon [5-7]. DIRS1-like elements are bounded by Inverted Terminal Repeats (ITRs) and harbor two Internal Complementary Regions (ICRs). The two ICRs located at the 3'-end of the element appear to overlap on a 3-bp motif called the circular junction. As the left ICR is inverse-complementary to the beginning of the left ITR so is the right ICR to the end of the right ITR, but the latter also appears complementary to an extension of the right ITR that is called the right Extension (rE) [1]. Given these unusual features, an integration model has been proposed [3,5] in which the ITRs' extremities match with their respective ICR. The junction of the two ITRs results in the formation of a rolling-circle intermediate of the element. The element integration then occurs by recombination between the 3-bp ITR junction sequence (complementary to the circular junction) and an identical sequence in the genome, which does not produce any target site duplications. Their unique structure distinguishes DIRS1-like retrotransposons from other YR-encoding elements, also known as the DIRS order [2] that includes also the Ngaro, Viper and PAT elements. The Ngaro and Viper retrotransposons are devoid of the MT domain and do not usually harbor ORF overlaps [6,8]. Elements from the PAT superfamily, the sister group of DIRS1-like retrotransposons, differ most prominently in their repeats. The PAT retrotransposons (PAT-like elements, TOC elements and kangaroo) are bounded by some "Split" Direct Repeats (SDRs) and can contain tyrosine recombinase-encoding regions in an inverted orientation [5].

Transposable elements have been found in all eukaryotic species investigated thus far [2]. However, depending on the superfamily or family of elements studied, they show different distributions among eukaryotes. For example, the Ty1/Copia, Ty3/Gypsy, LINEs, SINEs retrotransposons and the Tc1/Mariner transposons, have been detected almost ubiquitously [2,7,9-11]. The Penelope retrotransposons are also abundant in many animal species, but seem to be rare among plants, protists and fungi [12]. In contrast to this, the Maverick transposons (also called Polintons) have been characterized by a highly patchy distribution in diverse eukaryote species, but not in plants [13,14]. Until recently, bibliographic data and automatic annotations have revealed the presence of DIRS1-like retrotransposons only in 43 diverse eukaryote organisms (Table 1), mostly with a low diversity per species (up to four families in *Strongylocentrotus purpuratus *and three families in *Danio rerio *[1,5]) with the notable exception of *Xenopus tropicalis *(73 families deposited in Repbase [15]). They were not described in several well-studied groups (e.g., plants and mammals), and are absent from model organisms such as *Saccharomyces cerevisiae *and *Drosophila melanogaster*. The DIRS1-like retrotransposons appear widely distributed among decapod crustaceans [16]. These elements were previously detected using PCR approaches in 16 decapod species, including some shrimps, lobsters, crabs and galatheid crabs. The wide distribution among decapods and the continuous identification of elements in new species with the emergence of large-scale genome sequencing call into question their supposedly patchy distribution among eukaryote species.

**Table 1:** Survey of the eukaryote species in which DIRS1-like retrotransposons were previously detected

Higher taxon	Species	References
Actinistia	*Latimeria menadoensis*	Repbase^1^

	*Danio rerio*	[1,8]
	
	*Oncorhynchus mykiss*	GenBank (2006)
	
Actinopterygii	*Salmo salar*	GenBank (2006)
	
	*Takifugu rubripes*	[46]
	
	*Tetraodon nigroviridis*	[1]

Amoebozoa	*Dictyostelium discoideum*	[47]

	*Xenopus laevis*	[1]
	
Amphibia	*Xenopus tropicalis*	[1]

Cnidaria	*Nematostella vectensis*	[48]

Crustacea	*Daphnia pulex*	[25,24]
	
	16 decapod species	[16]

Dinoflagellata	*Perkinsus marinus*	GenBank (2010)

	*Arbacia punctulata*	[21]
	
Echinodermata	*Lytechinus variegates*	[8]
	
	*Strongylocentrotus purpuratus*	[1]

Hemichordata	*Saccoglossus kowalevskii*	GenBank (2010)

	*Apis mellifera*	[21]
	
	*Camponotus floridanus*	[27]
	
	*Glyptapanteles indiensis*	GenBank (2008)
	
Hexapoda	*Harpegnathos saltator*	[27]
	
	*Nasonia vitripennis*	GenBank (2007)
	
	*Solenopsis invicta*	[28]
	
	*Tribolium castaneum*	[21]

Mucoromycotina	*Phycomyces blakesleeanus*	[49]
	
	*Rhizopus oryzae*	[8]

Sauropsida	*Gopherus agassizii*	[5]

Urochordata	*Oikopleura dioica*	[50]

All the detected DIRS1-like elements, even in partial sequences, are reported here.

Notes: ^**1**^Repbase: version 14.06 (http://www.girinst.org/repbase/update/index.html).

We aim to determine the distribution of DIRS1-like retrotransposons among eukaryotes using an *in silico *approach. In the post-genome era, several automatic annotation tools have been developed to detect the presence of particular types of transposable elements in genomes. The conventional approaches are based on similarity searching using the RepeatMasker program [17]. However, transposable elements often correspond to ancient genome components. Many copies even within the same family appear fragmented and divergent in nucleotide sequences due to several punctual mutations, rearrangements, and insertions or deletions (indels). Similarity searching-based programs are efficient in identifying copies closely related to those previously reported in the library, but they often appear inefficient in detecting very divergent copies or unknown elements [18]. Other *in silico *approaches have been developed to detect particular types of elements. These programs, such as LTRharvest [19], are not based upon similarity searching but on specific signature searches (e.g., the nature of the termini and the presence of target site duplications). While some programs have been developed to detect LTR retrotransposons or transposons, none have been developed for DIRS1-like retrotransposons. Such a program might appear inefficient in identifying divergent DIRS1-like retrotransposons because the training dataset that is currently available for these elements remains too limited (only 18 reference elements with detectable ITRs for example). Some *de novo *approaches that detect more divergent transposable elements, such as RECON [20], have been developed to exhaustively report the content of repeated sequences within genomes. To identify a specific type of element, many investigations of this report must be performed, such as similarity searching. For the same reasons as those given for similarity searching-based methods, such approaches could appear inappropriate for studying the distribution of the DIRS1-like retrotransposons.

We hereby present a new computational approach specifically dedicated to the identification of DIRS1-like retrotransposons among genomes that we called ReDoSt. Our method is based on both the detection of the structure of these elements and on sequence similarity searches performed using alignment profiles designed on coding domains. It has the advantages of not considering the element copy number and of avoiding any preconception of the ITRs (length or sequence identity). With our method we analyzed 274 completely sequenced genomes, which allowed for a high coverage of eukaryotic diversity, especially plants and unikonts.

We have identified more than 4000 element copies that can be clustered into approximately 300 new families. We report the first DIRS1-like element copy number estimate among many genomes and we evaluate the diversity within the DIRS1-like superfamily. Their distribution appears wider than it was previously thought, especially in unikont species. Sequence analyses confirmed the presence of well-conserved DIRS1-like retrotransposons in 28 species, including at least 14 species that were not previously known to host such elements, and allowed us to define a more precise structure of the DIRS1-like retrotransposons, especially in their terminal repeats.

## Results and Discussion

### Identification of putative DIRS1-like retrotransposons in eukaryote genomes

To study the distribution of DIRS1-like retrotransposons among genomes, we developed a new computational tool that we call ReDoSt (Retrotransposon Domain and Structure). The element detection is mainly based on independent similarity searches against co-oriented and well-ordered RT-, MT- and YR-encoding domains within a single 10-kb genomic fragment (see Methods). So, the DIRS1-like copies detected with ReDoSt may be considered as well-conserved (*i.e*. with the simultaneous recognizable presence of these three characteristic domains), which suggests that they may still be active, or have moved only recently. Thus, relics and highly degenerate elements are not considered here.

Using ReDoSt, we identified 4310 copies of putative DIRS1-like elements distributed among 32 diverse species out of the 274 well-sequenced genomes tested (Table 2). A wide spectrum of eukaryote species is represented in which some taxa are characterized for the first time as harboring DIRS1-like retrotransposons. For example, we observed the first DIRS1-like elements in Mollusca (*Aplysia californica *and *Lottia gigantea*). Interestingly, DIRS1-like retrotransposons can be detected in all the species in two higher taxa, Actinopterygii and Mucoromycotina. ReDoSt was able to detect DIRS1-like elements in all species already described in the literature except those harbor in the honey bee *Apis melifera *genome. This discrepancy is due to the fact that this genome contains only remnant fragments of DIRS1-like elements that ReDoSt is unable to detect [21].

**Table 2:** Results of DIRS1-like retrotransposon detection and clustering

Higher taxon	Species	Copy number	Family number	Min	Max	Reference
	Danio rerio *	2091	14	1	1157	a
	
	*Gasterosteus aculeatus*	21	4	1	12	b
	
Actinopterygii	*Oryzias latipes*	6	1	-	-	c
	
	Takifugu rubripes *	7	1	-	-	d
	
	Tetraodon nigroviridis *	8	2	1	7	b

Amoebozoa	Dictyostelium discoideum *	16	1	-	-	[51]
	
	*Acantheamoeba sp.*	1	1	-	-	e

Amphibia	Xenopus tropicalis *	692	81	1	38	[52]

Annelida	*Capitella sp. I*	5	2	1	4	f

Blastocladiomycota	*Allomyces macrogynus*	21	6	1	10	b

Cephalochordata	*Branchiostoma floridae*	15	11	1	3	[53]

Chlorophyta	*Chlamydomonas reinhardtii*	11	5 (3)	1	4	[54]
	
	*Volvox carteri*	36	6 (4)	2	13	[55]

Cnidaria	Nematostella vectensis *	60	21 (1)	1	7	[48]

Crustacea	Daphnia pulex *	100	39	1	5	[56]

Echinodermata	Strongylocentrotus purpuratus *	4	4	-	-	e

Haptophytes	*Emiliana huxleyi*	1	1	-	-	f

Hemichordata	Saccoglossus kowalevskii *	240	8 (1)	1	175	e

Heterolobosea	*Naegleria gruberi*	7	6	1	2	[57]

	*Bombyx mori*	6	2	3	3	[58]
	
Hexapoda	Nasonia vitripennis *	37	18	1	4	e
	
	Tribolium castaneum *	1	1	-	-	e

Mucoromycotina	*Mucor circinelloides*	3	2	1	2	f
	
	Phycomyces blakesleeanus *	28	13	1	5	f
	
	Rhizopus oryzae *	24	11	1	4	[59]

Mollusca	*Aplysia californica*	39	7	2	10	b
	
	*Lottia gigantea*	44	22 (1)	1	5	f

Nematoda	*Caenorhabditis briggsae *^$^	1	1 (1)	-	-	g
	
	*Pristionchus pacificus *^$^	4	3 (3)	1	2	g

Petromyzontida	*Petromyzon marinus*	2	2	-	-	g

Sauropsida	*Anolis carolinensis*	775	42	1	319	b

Urochordata	Oikopleura dioica *	4	2	1	3	h

For each species, the number of sequences detected using ReDoSt and the number of families obtained with the MCL program are given. When they are informative, the minimum (Min) and the maximum (Max) numbers of sequences included in a family are provided. Species in which the presence of DIRS1-like elements was previously reported (cf. Table 1) are indicated with an asterisk. In the family number column, numbers in brackets indicate the number of families that we characterized as PAT-like elements. The two Nematoda species that comprise only PAT-like elements are indicated with a dollar. The clustering was performed on all sequences detected in the 32 species. The families shared by several species are represented several times in the table.

Notes: a: The zebrafish genome sequencing project at the Sanger Institute (http://www.sanger.ac.uk/Projects/D_rerio/) funded by the Wellcome Trust. b: The Broad Institute of Harvard and MIT ( http://www.broadinstitute.org/). c: The National Institute of Genetics and the University of Tokyo (http://medakagb.lab.nig.ac.jp/Oryzias_latipes/index.html). d: The Institute of Molecular and Cell Biology (http://www.fugu-sg.org/). e: The Baylor College of Medicine Human Genome Sequencing Center (http://www.hgsc.bcm.tmc.edu/project-species-x-organisms.hgsc). f: The U.S. Department of Energy Joint Genome Institute (http://www.jgi.doe.gov/genome-projects/). g: The Genome Institute at Washington University School of Medicine in St. Louis (ftp://genome.wustl.edu/pub/organism/). h: The Genoscope (http://www.genoscope.cns.fr/externe/GenomeBrowser/Oikopleura/).

As expected, the identified elements seem to be well-conserved. The length of the three detected domains appears highly constrained within the elements of a given genome. For example, in the Sauropsida *Anolis carolinensis *genome, almost all RT-, MT- and YR-encoding fragments have a length ranging from 360 to 380 bp, 300 to 320 bp, and 900 to 940 bp, respectively (Additional File 1). This pattern is present in most genomes, with the notable exception of *Saccoglossus kowalevskii*, which varies considerably in its domain length (Additional File 1), possibly because of multiple large fragment deletions.

Considering the repartition of the 4310 copies detected in 32 eukaryotes, the copy number per genome appears highly variable (Table 2), even within some of the higher taxa examined. In Actinopterygii, the low copy numbers detected in *Oryzias latipes, Takifugu rubripes, Tetraodon nigroviridis *and *Gasterosteus aculeatus *(6, 7, 8, and 21 copies, respectively) contrast with the 2091 copies identified in *D. rerio*. Conversely, in Mucoromycotina, *Mucor circinelloides *has ten times fewer copies than other related species. The copy number per genome is usually relatively low, illustrated by the fact that half of the species harbor fewer than 8 copies. Twelve species show between 10 and 60 copies and only 5 species harbor more than 100 copies (*D. pulex, S. kowalevskii, X. tropicalis, A. carolinensis *and *D. rerio*). This suggests that the more or less recent element activity is relatively low, resulting either from the inactivation of most genomic copies or from a strong regulation of the copy number. The loss of elements in some higher taxa or species could be facilitated by this low copy number. However, the relatively low copy number observed in genomes has to be conservative since only well-conserved copies are considered based on the three coding domains studied. For example, similarity searches on *Acantheamoeba *sp. allowed us to reveal 29 more degenerate sequences related to the unique element detected using ReDoSt (data not shown).

To our knowledge, the copy number has only been previously estimated in two genomes: the slime mold *Dictyostelium discoideum *and the crustacean *Daphnia pulex*. In *D. discoideum*, the previous copy number estimation of DIRS1-like retrotransposons suggested 40 full-size elements and around 200 incomplete copies [22]. Our detection tool results in the identification of 16 well-conserved copies. This result seems consistent with the previous estimation considering the difference in the methods used. The previous analysis estimated the copy number with quantitative Southern-blot experiments using the complete DIRS1-like sequence as a probe. For this reason it may detect more altered elements than our tool does. This is especially the case with the nested elements [23] that amplify the signal in Southern blots but are by default considered to be a unique copy by *in silico *ReDoSt analysis (see Methods). In *D. pulex*, the DIRS1-like copy number has been previously estimated at 218 [24], including only 19 intact copies (i.e., uncorrupted sequences and conserved ITRs) [25]. This estimation also seems consistent with our results (100 copies detected), as ReDoSt identifies well-conserved elements but is not limited to intact copies.

### The diversity of DIRS1-like retrotransposons

To study the diversity of the DIRS1-like elements, we use the MCL program to cluster into families all the sequences that were detected with ReDoSt as well as reference elements. The parameter values used to cluster in the MCL program were empirically estimated to discriminate each of the DIRS1-like families previously described (e.g., DrDIRS1, DrDIRS2 and DrDIRS3 in *D. rerio*). Based on the sequence identity, the clusters obtained on the reverse transcriptase-encoding sequences using the MCL program are considered to correspond to different DIRS1-like families. For example, the sequence identities among the largest cluster in *A. carolinensis *(319 sequences) range from 57% to 100%, with an average sequence identity of 81%. Such a relatively high nucleotide sequence divergence is similar to those observed in reverse transcriptases encoded by non-LTR retrotransposons and in some DNA transposases. The cluster number obtained in each genome reflects the diversity of DIRS1-like elements.

**Figure 2:**
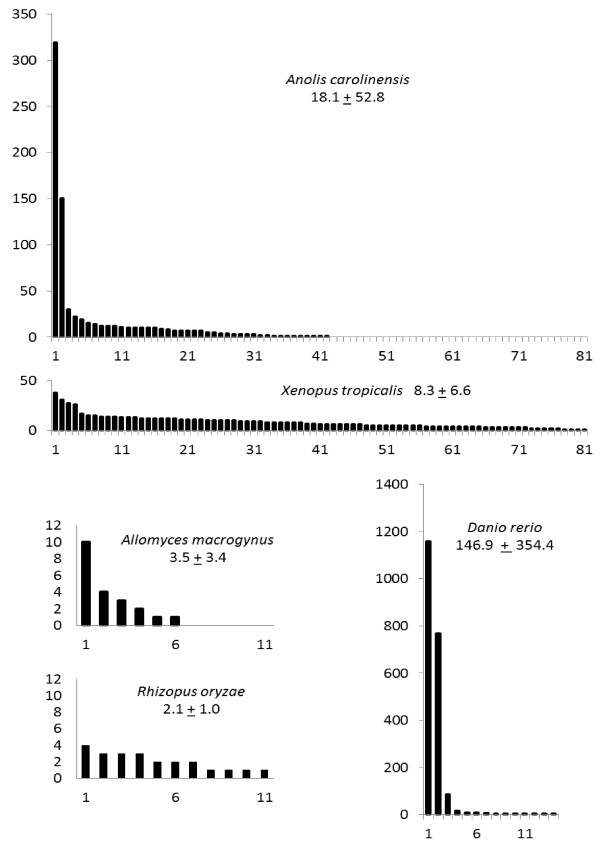
**Distribution of family size in five representative species**. Families are arranged along a gradient of decreasing size. For each species, mean family size and standard deviation are given. X-axis: family rank, Y-axis: number of elements in the family.

A total of 287 families were found distributed unevenly among the genomes of the 32 species examined (Table 2). Most of the families seem restricted to only one species with the notable exception of Mucoromycotina species for which several interspecific families are obtained. Some species show very low element diversity in comparison to their copy number. For example, all 16 copies detected in *D. discoideum *grouped into a single family. On the other hand, few species show very high element diversity. For example, *S. purpuratus *harbors 4 copies distributed among 4 families. Likewise, the 14 copies of *B. floridae *are split into 11 families. The distribution of copy number per family shows two major profiles according to species (Figure 2 and Additional File 2). Comparing the two vertebrate species *X. tropicalis *and *A. carolinensis*, both of which harbor high copy and family numbers, the Western clawed frog contains families almost equal in size whereas the lizard contains two families that together include 64% of the copies. The two fungi *Rhizopus oryzae *and *Allomyces macrogynus *have only about 20 copies, which are well distributed in *R. oryzae *while half of the copies of *A. macrogynus *belong to one family. Finally, in *D. rerio*, which harbors the highest copy number, 96% of the 2091 copies belong to just three families (1157 and 767 copies for DrDIRS1 and DrDIRS2, respectively). Such a distribution with a high copy number restricted to few families could be related to bursts of transposition. Bursts of DIRS1-like element activity are also suspected in *S. kowalevskii *(the SkoDIRS1 family alone accounts for 175 of the 240 copies identified) and in *A. carolinensis *(AcDIRS1 and AcDIRS2 families together harbor more than 60% of the different copies).

### Phylogenetic analysis of DIRS1-like retrotransposons

**Figure 3:**
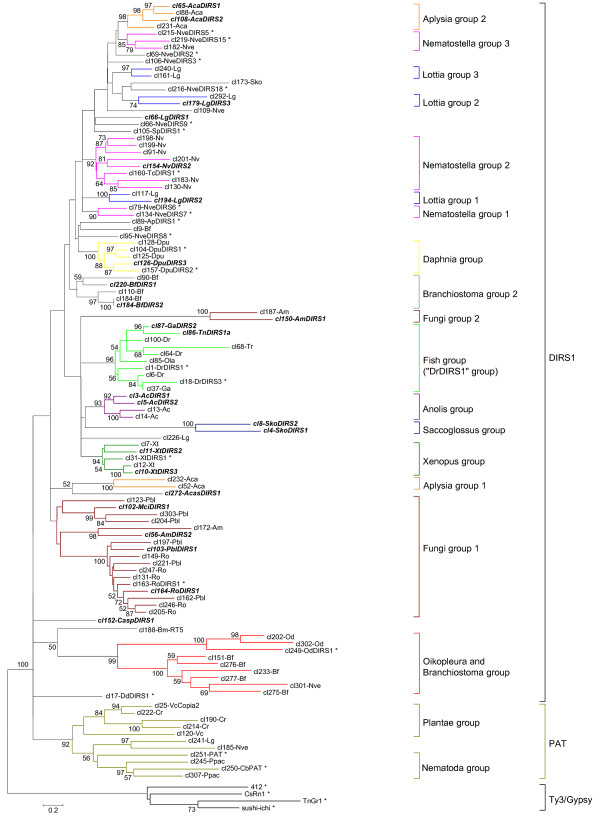
**Rooted phylogenetic tree based on the *pol *amino acid sequences of the DIRS1-like families identified**. Distances are calculated with JTT parameter model plus gamma distribution's correction for amino acids. The tree is constructed using the Neighbor Joining method and pairwise deletion of gaps option included in MEGA5.0 software. When possible, one representative copy sequence that required only minor corrections for each family was integrated into our analysis. Reference elements are labeled with an asterisk and clusters that correspond to an element annotated in this study are written in bold italics. If a reference element was included in a family, this sequence was chosen to represent the family. In the cases of *Anolis carolinensis, Daphnia pulex *and *Xenopus tropicalis*, species that show a high family number, only four or five of their most abundant families were integrated. Ty3/Gypsy element sequences were used as outgroups according to the close relationships of their reverse transcriptase and RNase H domains with those of DIRS1-like and PAT retrotransposons. Support for individual groups was evaluated with non-parametric bootstrapping using 100 replicates. Only bootstrap node values over 50% are represented.

To infer the relationships among the various members of DIRS1-like superfamily, we constructed a phylogenetic tree (Figure 3) based on an alignment of amino acid *pol *region sequences (214 sites). This phylogenetic tree contains 114 sequences, including a representative sequence of each family that has at least one uncorrupted copy, 23 DIRS1-like or PAT-like reference elements and 4 Ty3/Gypsy elements used as outgroups. Preliminary analysis of the three genomes that present high family numbers (42 families in *A. carolinensis*, 39 in *D. pulex*, and 81 in *X. tropicalis*) has shown that all of the elements from a given species cluster together into a monophyletic group (data not shown). For these species, only representative elements from the 4 or 5 largest families were included in the phylogenetic analysis. In contrast to previous analyses on much smaller datasets, the monophyly of DIRS1-like elements is not supported in the present study (bootstrap support lower than 75%). Such a pattern could be an artifact of a dataset that is too large and includes divergent elements. Alternatively, it might suggest that the PAT elements belong to the DIRS1-like superfamily, representing a peculiar group because of their structure. Many well-supported groups can be identified within the DIRS1-like elements. In many cases, the elements from a given species form a monophyletic group (e.g., elements from *Nasonia vitripennis, D. pulex *or *A. carolinensis*). However, some species harbor elements from two or three different groups (e.g., two and three element groups in *A. californica *and *L. gigantea*, respectively). In the same way, each group usually integrates elements from the same species or from a few closely related ones. For example, all the elements identified in fishes belong to one group called DrDIRS1 [21]. Likewise, the fungi group 1 comprises most of the elements identified in fungi, a result that confirms the close relationships between most fungi DIRS1-like elements revealed by the MCL analysis. Despite the difficulty in resolving the relationships among the different DIRS1-like groups, the monophyletic groups comprising only elements from a species or related species, the tree topology appears absent of clear evidence of horizontal transfer.

### Discriminating the PAT-like sequences included in the final dataset

The PAT-like retrotransposons are the sister group of DIRS1-like elements and show a similar structure with the exception of their termini [6]. To discriminate the putative PAT-like elements retained by ReDoSt, 5 PAT-like reference sequences were included during the clustering process and the phylogenetic analysis (Figure 3). This allowed us to determine that 11 families correspond to PAT-like retrotransposons (Table 2). This includes 6 families from the chlorophytes (*Chlamydomonas reinhardtii *and *Volvox carteri*), 3 families from the nematodes (*Caenorhabditis briggsae *and *Pristionchus pacificus*), one family from *L. gigantea*, and one shared by *Nematostella vectensis *and *S. kowalevskii*.

The presence of DIRS1-like retrotransposons is confirmed in 25 species, but still remains uncertain in *Emiliana huxleyi, Petromyzon marinus, Naegleria gruberi, P. pacificus, V. carteri *and *C. reinhardtii*. Elements from these species do not cluster with any reference elements and their sequences harbor too many frameshifts or indels to be included in our phylogenetic analysis. For these elements, we checked the presence of DIRS1-like elements using similarity searches using the TBLASTX program [26] and the Repbase database that we previously re-annotated for the DIRS1-like and PAT elements (data not shown). A family was assigned to the DIRS1-like element superfamily under the two conditions: (i) an E-value lower than 1e-20 with at least one DIRS1-like reference element; and (ii) a minimum difference between the best E-values obtained with DIRS1-like and PAT reference elements of 1e-10. Under these criteria, the presence of DIRS1-like retrotransposons could be confirmed in *V. carteri, P. marinus *and *N. gruberi*, but remains uncertain in *C. reinhardtii *and *E. huxleyi*, whereas the element detected in *P. pacificus *appears to be a PAT-like retrotransposon. So, 30 of the 32 species revealed by ReDoSt are now considered as harboring DIRS1-like retrotransposons and the two remaining posses in fact only PAT elements.

### Distribution of DIRS1-like elements among eukaryotes

**Figure 4:**
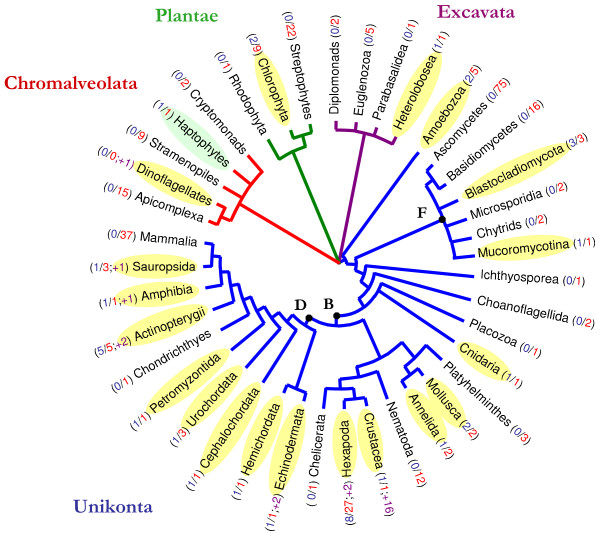
**Distribution of DIRS1-like elements among the eukaryote groups tested**. Species phylogeny was redrawn from [42-45]. Chromalveolate species are shown with red branches, excavates with purple branches, plants with green branches, and unikonts with blue branches. Groups in which DIRS1-like elements were detected and confirmed are shaded in yellow. Groups in which the presence of DIRS1-like element remains uncertain are shaded in green. In each group, we include in parentheses the number of species harboring DIRS1-like sequences (in blue) compared to the number of species analyzed (in red), as well as the number of species not screened in this study in which DIRS1-like retrotransposons were previously reported (in purple). B: Bilateria, D: Deuterostomia, F: Fungi.

DIRS1-like retrotransposons are now described in 61 diverse eukaryote species (Figure 4), including 14 species in 8 higher taxa newly characterized using ReDoSt: annelids, blastocladiomycetes, cephalochordates, chlorophytes, heteroloboseans, molluscs, petromyzontids and sauropsids. The DIRS1-like element distribution does not seem to be as patchy as it was previously described. Sixteen of the 28 unikont groups tested revealed the presence of these elements, indicating a wide distribution. This distribution could be shown to be wider in the near future since seven of the unikont groups apparently devoid of DIRS1-like elements are currently represented by only one or two completely sequenced genomes. Conversely, four other unikont groups seem to be clearly devoid of DIRS1-like elements. Despite a high number of completely sequenced genomes and diverse taxa tested, no well-conserved copies could be identified in any ascomycetes (75 species), basidiomycetes (16 species), nematodes (12 species) or mammals (37 species). A specific loss of DIRS1-like elements in Mammalia during evolution is the most probable cause of their absence when one takes into consideration their wide distribution in Unikonta, especially Deuterostomia. Outside of unikonts, DIRS1-like retrotransposons appear infrequently, observed in only three groups, even though most groups are represented by relatively few species.

Various distributional patterns can currently be observed among eukaryotes. On a large phylogenetic scale, we make two observations: (i) a wide distribution of DIRS1-like elements among groups such as deuterostomes, with the detection of copies in a wide range of higher taxa from Echinodermata to Sauropsida; and (ii) a large repartition of the DIRS1-like elements observed in certain taxa despite a lack of detection in closely related taxa. In fungi, all three Mucoromycotina genomes were found to harbor DIRS1-like elements, whereas none could be detected in Ascomycota and Basidiomycota. On a smaller phylogenetic scale (i.e., within a higher taxon), the distribution again appears to be taxon-dependent with three distinguishable patterns. As described above, some groups seem to possess no DIRS1-like retrotransposons (e.g., mammals and streptophytes). Second, a large repartition of DIRS1-like elements was observed in some groups such as in Actinopterygii and Mucoromycotina (detection in all 5 and 3 genomes tested, respectively). Finally, a sparser distribution of DIRS1-like elements was observed in yet other groups. Only 3 of the 22 hexapod species tested harbor well-conserved elements. However, this heterogeneous distribution could result in part from a sampling bias. We observed a lack of elements in some overrepresented taxa, such as Diptera (absence of detection in 16 *Drosophila *species tested), and an abundance in others, such as Hymenoptera (in three wasp and five ant species). Indeed, we used ReDoSt to analyze the recently released ant genomes, all of which harbor DIRS1-like elements. Five copies were found in *Camponothus floridanus*, 22 in *Pogonomyrmex barbatus*, 37 in *Harpegnathos saltator*, 41 in *Linepithema humile*, and 57 in *Solenopsis invicta *[27-31].

The previous though that DIRS1-like retrotransposons are uncommon among eukaryotes appears to be strongly biased considering that ascomycetes, mammals and green plants, which are devoid of elements, represent more than 55% of the sequenced genomes. DIRS1-like elements do not appear as ubiquitous as Ty1/Copia and Ty3/Gypsy retrotransposons but their distribution among eukaryotes appears more comparable to the Penelope element distribution [12,13]. Despite their loss in several lineages, the phylogenetic analysis and the distribution of DIRS1-like elements in a very broad range of unikonts indicate that their genomic invasion occurred early in unikont evolution; at least prior to the Bilateria radiation but probably before if we take into account the presence of DIRS1-like retrotransposons in Amoebozoa and Fungi (Figure 4). This primary invasion could be found to have occurred earlier in evolution if the presence of DIRS1-like elements is confirmed in Excavata, Plantae and Chromoalveolata. Though our results unequivocally indicate the presence of DIRS1-like elements in Unikonta, we must be cautious in our estimation of their real distribution in Excavata and Plantae because most of the copies identified in these taxa harbor too many indels and frameshifts in the repeated sequence structures to be studied and for them to be included in the phylogenetic analysis. The presence of DIRS1-like elements in these species is only supported by similarity search analyses.

The absence of DIRS1-like elements in several groups may reflect their differential success in adapting to different host species and/or a propensity for stochastic loss during evolution. Nevertheless, this absence has to be confirmed in the future by investigations of deleted DIRS1-like copies in these genomes. The detection of deleted copies in an apparently "unoccupied" species would be evidence of the previous existence of well-conserved DIRS1-like elements.

### In-depth characterization of new DIRS1-like elements

To describe the diversity within the DIRS1-like superfamily, we detailed the structure of 28 new elements, most of which represent high copy number families or species newly characterized for the presence of such retrotransposons (e.g., *A. californica *and *L. gigantea*). Several features of DIRS1-like retrotransposons are presented in Table 3, such as their length, the presence of a long ORF overlap, and the structure of their repeats. The length of DIRS1-like retrotransposons appears variable between the 28 elements from 3974 bp in AcasDIRS1 (*Acantheamoeba *sp.) to 6283 bp in SkoDIRS2 (*S. kowalevskii*), with an average length of 5160 bp. In-depth annotation including the positions of the repeated sequences and several conserved motifs is provided in Additional File 3. The *pol *motifs seem to be highly conserved, especially the 'YL/IDD' motif that is conserved in 25 of the 28 annotated elements. The 'HSTR' tyrosine recombinase motif appears more variable (only harbored by 13 of the 28 elements). For example, AmDIRS2 and MciDIRS1 harbor an 'SDLK' and 'LCPV' sequence, respectively. This suggests that the catalytic tyrosine recombinase-encoding domain sequence could be less constrained than the *pol *sequence. Twenty-three of the elements begin and end with a trinucleotide NTT, most frequent being ATT (Table 3). Only the AmDIRS1 from *A. macrogynus *begins and ends with an uncommon GC-rich motif. In almost all elements, this trinucleotide appears complementary to the 3-bp circular junction. Evidence of long ORF overlaps was found in half of the 28 DIRS1-like elements, which seems to depend on host species (e.g., evidence in the five elements from Fungi and none in Mollusca).

**Table 3:** Annotation of 28 DIRS1-like retrotransposons

									Termini	size		ICR
								
Element	Host		Size	start	end	circular	long ORF	lE	divergent	conserved	rE	size
						junction	overlap		ITR	ITR		
AcDIRS1	*A. carolinensis*	Sauropsida	5938	CTT	CAT	AAG	No	-	26	140	26	40-58

AcDIRS2	*A. carolinensis*	Sauropsida	5997	TTT	TGT	AAA	Yes	-	26	136	28	39-60

AcaDIRS1	*A. californica*	Mollusca	5222	ATT	ATT	AAT	No	-	36	32	48	45-88

AcaDIRS2	*A. californica*	Mollusca	5808	ATT	ATT	AAT	No	-	34	36	48	44-88

AcasDIRS1	*A. castellanii*	Amoebozoa	3974	GTT	CTT	AAG	No	12	22	84	-	52-36

AmDIRS1	*A. macrogynus*	Blastocladiomycota	4801	CGG	GCG	CG	Yes	64	33	123	-	110-39

AmDIRS2	*A. macrogynus*	Blastocladiomycota	5713	ATT	AAT	AAT	Yes	14	9	193	75	24-86

BfDIRS1	*B. floridae*	Cephalochordata	4949	TTG	TTG	CAA	Yes	-	23	118	45	60-83

BfDIRS2	*B. floridae*	Cephalochordata	5269	TTA	TTT	AA	Yes	-	19	103	40	33-71

BmDIRS1	*B. mori*	Hexapoda	4870	ATT	ATA	AAT	Yes	-	16	155	30	40-62

CaspDIRS1	*Capitella sp.*	Annelida	4994	ATT	ATT	AAT	Yes	nd	nd	nd	nd	nd

DIRS1-2	*D. discoideum*	Amoebozoa	3793	TTA	TTA	TAA	Yes	-	12	304	24	26-59

DpuDIRS3	*D. pulex*	Crustacea	5195	ATT	ATT	AAT	No	-	24	170	50	38-89

GaDIRS1	*G. aculeatus*	Actinopterygii	5322	GTT	GTT	AAC	Yes	-	19	148	27	46-56

GaDIRS2	*G. aculeatus*	Actinopterygii	5782	GTT	GTT	AAC	Yes	-	21	144	26	38-57

LgDIRS1	*L. gigantea*	Mollusca	4680	ATT	ATT	AAT	No	-	35	31	48	39-86

LgDIRS2	*L. gigantea*	Mollusca	5036	AAT	AAT	ATT	No	-	34	141	46	46-67

LgDIRS3	*L. gigantea*	Mollusca	5133	ATT	ATT	AAT	No	-	30	36	49	42-86

MciDIRS1	*M. circinelloides*	Mucoromycotina	4402	ATT	ATT	AAT	Yes	38	8	107	11	75-42

NvDIRS2	*N. vitripennis*	Hexapoda	4849	TAA	TAA	TTA	No	-	25	62	35	41-73

NveDIRS6b	*N. vectensis*	Cnidaria	4142	ATT	AAT	AAT	No	-	30	118	49	40-82

PblDIRS1	*P. blakesleeanus*	Mucoromycotina	4315	ATT	ATT	AAT	Yes	28	20	94	9	58-39

RoDIRS1	*R. oryzae*	Mucoromycotina	4274	ATT	ATT	AAT	Yes	28	14	102	9	59-44

SkoDIRS1	*S. kowalevskii*	Hemichordata	6052	ATT	ATT	AAT	No	-	16	202	30	67-63

SkoDIRS2	*S. kowalevskii*	Hemichordata	6283	GTT	GTT	AAC	No	-	15	142	27	63-65

TnDIRS1a	*T. nigroviridis*	Actinopterygii	5931	GTT	GAT	AAC	Yes	-	21	146	27	36-57

XtDIRS2	*X. tropicalis*	Amphibia	5571	TTT	TAT	AAA	Yes	-	20	102	30	43-61

XtDIRS3	*X. tropicalis*	Amphibia	6196	TTT	TTT	AAA	Yes	-	11	123	34	41-65

The element size and trinucleotide sequences beginning and ending the element complementary to the circular junction are given for each manually annotated DIRS1-like element. Evidence of long ORF overlap is also indicated. The lengths of the different parts of the termini (the divergent and the conserved ITRs, lE and rE) as well as those of the ICRs are reported. nd: not determined because CaspDIRS1 corresponds to a chimeric sequence. Each newly identified element has been submitted to Repbase.

**Figure 5:**
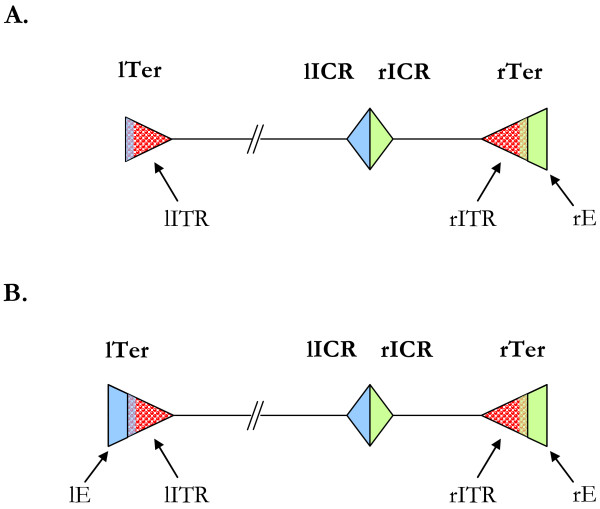
**Structure of repeated sequences observed within DIRS1-like retrotransposons**. The left (lTer) and right (rTer) termini as well as the left (lICR) and right (rICR) ICRs are represented by triangles. ITRs are represented by bubbles. Blue items correspond to the complementary lTer and the lICR sequences. Green items correspond to the complementary rTer and the rICR sequences. Colored parts of the lTer and rTer that do not overlap red bubbles symbolize the lE and rE sequences. A: Element harboring only one extension (here the rE sequence). In this case, the lICR is only complementary to the left ITR (lITR); B: Elements harboring both the lE and rE sequences.

**Figure 6:**
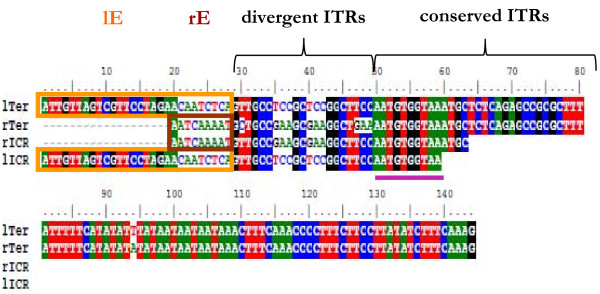
**Alignment of the nucleotide sequences of RoDIRS1 element termini and ICRs**. In this alignment the left terminus (lTer) and the right ICR (rICR) sequences are represented with the inverse complementary sequences of the right terminus (rTer) and the left ICR (lICR) sequences. The left and right extensions (lE and rE) of the terminal regions are indicated by orange and brown boxes. The portion called "divergent ITRs" represents the part of the ITRs that is mostly complementary to the corresponding ICRs. The region that is conserved among the four different sequences is underlined in purple.

Previous studies have outlined the structure of DIRS1-like retrotransposons, especially the nature of their termini, which complement the Internal Complementary Regions (ICRs), and the presence of a right Extension sequence (rE) [3]. Looking in detail at the repeated sequences "lITR-lICR-rICR-rITR-rE" in these elements allowed us to reveal a rather more complex structure (Figure 5). Whereas previous studies only allowed the description of a rE sequence, we have characterized an equivalent left Extension sequence (lE) at the 5'-end of some elements, which is only complementary to the left ICR. The identification of this additional lE sequence does not challenge the replication model that proposes a rolling-circle intermediate. This intermediate is produced by the 3-bp circular junction that corresponds to the overlap of the two ICRs complementary to the 5'- and 3'- ends of the element [3-5]. All elements harbor at least one extension, and, like DIRS1, most elements contain only a rE. The lE region has only been detected in fungi and amoebozoa species. Two elements show only a lE (AcasDIRS1, AmDIRS1) and four other elements harbor the two extensions (e.g., AmDIRS2, MciDIRS1). We hereby propose to redefine the fine structure of the DIRS1-like element's termini (Figures 5 and 6). In this study we call the left and right termini (lTer and rTer) the assembly of the two components: the ITRs and their respective potential extension (lE or rE). The lE and rE regions are considered the external sequences of the termini that are only complementary to their respective ICR sequences (theoretically 100% sequence identity). The ITRs are defined as the parts of these terminal sequences that are mostly complementary to each other. On a smaller scale, two parts can be distinguished within these ITRs (Figure 6). In the conserved ITR part, the two ITRs are strictly complementary to each other. In the divergent ITR part, the two ITR sequences are mostly constrained by their respective ICR and remain only partially complementary to each other, with a sequence identity that varies from 50% to 85%. ITR length appears highly variable among the different elements, ranging from 66 bp (LgDIRS1) to 316 bp (DIRS1-2). Likewise, the length of the ICRs varies between 85 bp for the sum of the two ICRs in AcasDIRS1 and 130 bp in AcaDIRS1. The right extensions vary from 9 bp to 75 bp (being apparently shorter in the presence of a lE). In most cases, the sizes of the various repeats are conserved among the different elements from the same species (e.g., among AcDIRS1 and AcDIRS2, or AcaDIRS1 and AcaDIRS2). The conserved ITR usually represents the largest part of the ITR, ranging from 31 bp to 304 bp (Table 3), whereas the divergent ITR is often small, ranging from 9 bp to 36 bp. However, in some elements from molluscs both parts have about the same size. Interestingly, the boundary between these two ITR parts is composed of a short sequence of at least 10 nucleotides that is conserved in two ITRs and two ICRs (Figure 6), which may be involved in the formation of the circular intermediate of the element before its integration.

## Conclusions

In this study, we developed a new computational tool, ReDoSt, allowing us to describe more precisely the distribution of DIRS1-like retrotransposons as well as their diversity among eukaryote genomes. These elements appear more continuously distributed than previously though, with 8 new higher taxa characterized to harbor these elements (e.g. Mollusca) and 14 new eukaryote species, giving a total of 61 species containing DIRS1-like elements in their genome. The current understanding of the distribution of DIRS1-like elements in Eukaryota, and especially Unikonta, suggests the presence of DIRS1-like elements in the last common ancestor of eukaryotes. Whereas some higher taxa seem clearly devoid of well-conserved DIRS1-like retrotransposons (e.g., ascomycetes, mammals and streptophytes), these elements appear highly conserved in some other higher taxa, such as Actinopterygii and Mucoromycotina. Now that a large diversity of elements within the DIRS1-like superfamily (around 300 different families) have been characterized, it is possible to screen sequence datasets for the presence of DIRS1-like elements using more conventional approaches like RepeatMasker. This large diversity allowed us to study the phylogenetic relationships within the DIRS1-like superfamily in which the different groups appear related to the host species. All of the elements included in the phylogenetic analysis as well as the subset of 28 annotated elements were used to define two new alignment profiles for each of the three characteristic domains of the DIRS1-like retrotransposons: reverse transcriptase, methyltransferase and tyrosine recombinase. These profiles could be used in further studies or in future automatic annotation of transposable elements within genomes (Additional file 4).

## Methods

### Data collection

The 274 complete or draft genomic sequences were downloaded from eight different databases: the DOE Joint Genome Institute (http://www.jgi.doe.gov/), the Broad Institute of MIT and Harvard (http://www.broadinstitute.org/), the Human Genome Sequencing Center at the Baylor College of Medicine (http://www.hgsc.bcm.tmc.edu/), the Genome Center at Washington University (http://genome.wustl.edu/), the National Center for Biotechnology Information (http://www.ncbi.nlm.nih.gov/), the Wellcome Trust Sanger Institute (http://www.sanger.ac.uk/), Genoscope (http://www.genoscope.cns.fr/spip/) and FlyBase (http://flybase.org/). Five additional hymenopteran genomes were obtained from the Fourmidable database [29]. A complete list of all the genomes analyzed in this study and their sources is given in Additional file 5. Species were selected only if their genome is larger than 10 Mb with sequence coverage sufficient to represent their entire genome, which was labeled as complete or draft by the corresponding sequencing center or by the GOLD database (http://www.genomesonline.org/). Reference element sequences that were used in the alignment profile design, MCL clustering, and phylogenetic analysis correspond to the DIRS1-like sequences that we could access in GenBank, Retrobase (http://biocadmin.otago.ac.nz/fmi/xsl/retrobase/home.xsl) and Repbase Update database version 14.06 (http://www.girinst.org/repbase/update/index.html).

### Identification of DIRS1-like retrotransposons

**Figure 7:**
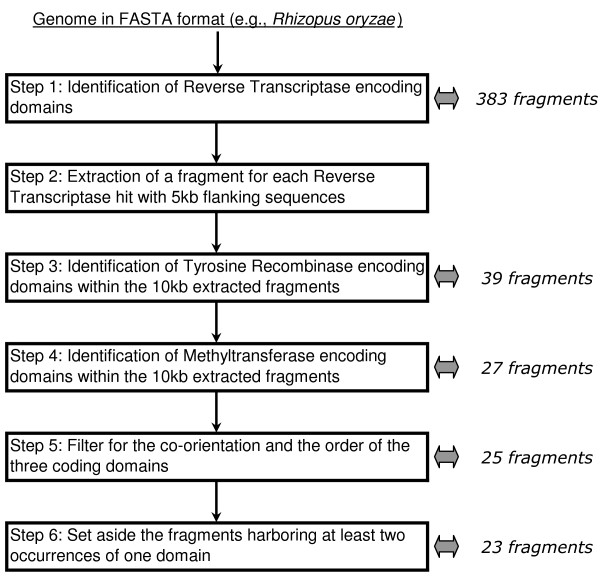
**ReDoSt pipeline developed in this study for the identification of DIRS1-like retrotransposons**. To assess the efficiency of each step of the pipeline, we detailed the number of fragments retained after each step for the genome of the fungus *Rhizopus oryzae.*

We propose a new computational tool for DIRS1-like retrotransposon identification, ReDoSt (Additional file 4, updates available at http://wwwabi.snv.jussieu.fr/public/ReDoSt/), based on both similarity searches of domains and their organization in the element structure. The similarity searches were performed using the RPS-BLAST and PSI-BLAST programs [32] with an E-value cutoff of 0.01 and specific alignment profiles for each domain. This method, in comparison with BLAST or RepeatMasker approaches, may be more permissive and thus allow for the identification of more divergent elements. For example, using this method we identified 21 DIRS1-like copies in the *A. macrogynus *genome, whereas only 16 well-conserved elements (i.e. simultaneous detection of the RT, MT and YR domains) were detected using RepeatProteinMask and the RepeatPeps library (included in the RepeatMasker package). We used three different profiles whose positions within the element are shown in Figure 1. For the RT-encoding domain, we used the alignment profile 'cd03714' (118 amino acids, Conserved, Domain Database, http://www.ncbi.nlm.nih.gov/). For the remaining two encoding domains, we used two specific alignment profiles (282 and 93 amino acids for the YR and MT profiles, respectively) that we designed using DIRS1-like reference element alignments (Additional file 4, http://wwwabi.snv.jussieu.fr/public/ReDoSt/). Our automatic detection tool is composed of six main steps (Figure 7): (1) Identification of all putative reverse transcriptase-encoding fragments within the genome; (2) Extraction of each genomic hit with 5-kb flanking sequence on both sides because all DIRS1-like elements described to date are less than 6 kb in length; Within each genomic fragment retained, (3) tyrosine recombinase-encoding domain search and (4) methyltransferase-encoding domain search; (5) After obtaining the 10-kb contigs that harbor the three characteristic domains (RT, YR and MT) of DIRS1-like retrotransposons, we checked the co-orientation and the order of these domains to discriminate other types of YR-encoding retrotransposons (e.g., Ngaro and PAT elements); (6) Finally, fragments that harbor at least two occurrences of the same domain were set aside for copy number estimation, sequence alignments, and supplementary investigations required to determine from which rearrangements (duplications or insertions) they are derived. Such a fragment has then been considered a single copy of DIRS1-like element in copy number estimation. We repeatedly observed a bottleneck between the first and fourth steps for all of the genomes tested (the example of *R. oryzae *results given in Figure 7). We chose to be less stringent in the first step by using an alignment profile designed using a large diversity of elements, one third represented by other types of tyrosine recombinase-encoding retrotransposons as well as one Gypsy element. As a consequence, many reverse transcriptase-encoding fragments identified may belong to other retrotransposon superfamilies. Analyses were performed on an iDataPlex Linux system (CPU 2.53 GHz, 3 GB memory).

### Sequence analysis

Families of DIRS1-like elements were identified by clustering all the nucleotide reverse transcriptase-encoding fragment sequences detected in the 32 species with the MCL program (http://www.micans.org/mcl/, [33]). Reference elements previously described and/or deposited in Repbase version 14.06 were also added to the dataset. This method was used in previous studies on IS transposons [34,35]. An E-value cutoff of 0.01 was used for the initial BLASTN search. An inflation factor of 1.2 was computed to cluster sequences. These values are effective at least in splitting elements of different previously defined DIRS1-like families (e.g., DrDIRS1, DrDIRS2 and DrDIRS3 in *D. rerio *[5]). Because clustering results can depend on the dataset used, we tested two different approaches: an independent clustering of the elements within each tested genome and a global clustering of all elements from all species. Similar results were obtained regardless of the approach used (data not shown), suggesting that the clusters obtained are well-supported.

To perform the element annotation, we preferentially selected elements from species in which DIRS1-like retrotransposons were not previously reported or from families showing high copy number. The repetitive structures (ICRs and ITRs) were detected using UGENE (http://ugene.unipro.ru/index.html). When several copies of a family were available for one species, the boundaries of the ITRs were manually analyzed and detection of the flanking regions in multiple nucleotide sequence alignments carried out using MUSCLE [36]. To check the presence of ORF overlaps, we used the ORF Finder tool (http://www.ncbi.nlm.nih.gov/projects/gorf/).

For phylogenetic analyses, a sequence from each family was included that required none or only minor corrections in its *pol *sequence (no large indels or multiple frameshifts). The amino acid *pol *sequence multiple alignments were performed with MUSCLE and ambiguously aligned sites were removed using Gblocks [37]. Phylogenetic analyses were conducted using neighbor-joining (NJ) method and the pairwise deletion option of the MEGA5.0 software [38]. The best-fit model, the JTT model [39] with gamma distribution, was selected with Topali2 software [40] and support for individual groups was evaluated with non-parametric bootstrapping [41] using 100 replicates.

## List of abbreviations used

ICR: Internal Complementary Region; ITR: Inversed Terminal Repeat; lE: left Extension; LINE: Long Interspersed Element; LTR: Long Terminal Repeat; lTer: left Terminus; MT: MethylTransferase; rE: right Extension; RH: RnaseH; RT: Reverse Transcriptase; rTer: right Terminus; SDR: "Split" Direct Repeat; SINE: Short Interspersed Element; YR: Tyrosine Recombinase.

## Authors' contributions

EB and IRG conceived and coordinated the study. MP and IRG carried out the *in silico *element detection. MP performed the clustering and phylogenetic analyses. EB and DH carried out the element annotations. MP drafted the paper. All authors read and approved the final manuscript.

## Description of additional data files

The following additional data are available with the online version of this paper. Additional data file 1 contains two histograms representing the distribution of the domain sizes for the elements detected in *A. carolinensis *and *S. kowalevskii*. Additional data file 2 contains histograms of the distribution of family size in several species. Additional data file 3 provides a table listing features of the 28 DIRS1-like annotated elements. Additional data file 4 is a mini-website providing an access to the ReDoSt pipeline, to the different alignments profiles and to the DIRS1-like sequences used to design them. Additional data file 5 is a list reporting the data source for all species tested.

## Supplementary Material

Additional file 1 **Domain size distributions for the elements detected in *A. carolinensis *(A) and *S. kowalevskii *(B)**. The histogram represents the number of element domains detected (y-axis) as a function of their length (x-axis). The reverse transcriptase fragments are represented in blue, the methyltransferase fragments in red, and the tyrosine recombinase fragments in yellow. Click here for file

Additional file 2 **Distribution of family size**. Families are arranged along a gradient of decreasing size. For each species, mean family size and standard deviation are given. X-axis: family rank, Y-axis: number of elements in the family. Click here for file

Additional file 3 **Annotation of the 28 DIRS1-like elements described**. For each element, positions of the repeated sequences within elements, the tyrosine recombinase and *pol *conserved motifs (reverse transcriptase (RT), RNase H (RH) and methyltransferase domains), and the end of the putative *pol *region are reported. The position of each element within the genome sequences is also provided. Click here for file

Additional file 4 **ReDoSt pipeline and alignment profiles used in this study**. Click here for file

Additional file 5 **List of all species tested**. For each species, the acronym used during the study and the data source website are indicated. Click here for file

## References

[B1] Goodwin TJ Poulter RT The DIRS1 group of retrotransposons Mol Biol Evol 2001 18 2067 2082 11606703 10.1093/oxfordjournals.molbev.a003748

[B2] Wicker T Sabot F Hua-Van A Bennetzen JL Capy P Chalhoub B Flavell A Leroy P Morgante M Panaud O Paux E SanMiguel P Schulman AH A unified classification system for eukaryotic transposable elements Nat Rev Genet 2007 8 973 982 10.1038/nrg2165 17984973

[B3] Cappello J Handelsman K Lodish HF Sequence of Dictyostelium DIRS-1: an apparent retrotransposon with inverted terminal repeats and an internal circle junction sequence Cell 1985 43 105 15 10.1016/0092-8674(85)90016-9 2416457

[B4] Curcio MJ Derbyshire KM The outs and ins of transposition: from mu to kangaroo Nat Rev Mol Cell Biol 2003 4 865 877 14682279 10.1038/nrm1241

[B5] Poulter RTM Goodwin TJD DIRS-1 and the other tyrosine recombinase retrotransposons Cytogenet Genome Res 2005 110 575 588 10.1159/000084991 16093711

[B6] Lorenzi HA Robledo G Levin MJ The VIPER elements of trypanosomes constitute a novel group of tyrosine recombinase-enconding retrotransposons Mol Biochem Parasitol 2006 145 184 194 10.1016/j.molbiopara.2005.10.002 16297462

[B7] Llorens C Muñoz-Pomer A Bernad L Botella H Moya A Network dynamics of eukaryotic LTR retroelements beyond phylogenetic trees Biol Direct 2009 4 41 10.1186/1745-6150-4-41 19883502 PMC2774666

[B8] Goodwin TJD Poulter RTM A new group of tyrosine recombinase-encoding retrotransposons Mol Biol Evol 2004 21 746 759 10.1093/molbev/msh072 14963102

[B9] Kramerov DA Vassetzky NS Short retroposons in eukaryotic genomes Int Rev Cytol 2005 247 165 221 16344113 10.1016/S0074-7696(05)47004-7

[B10] Ohshima K Okada N SINEs and LINEs: symbionts of eukaryotic genomes with a common tail Cytogenet Genome Res 2005 110 475 490 10.1159/000084981 16093701

[B11] Piskurek O Jackson DJ Tracking the Ancestry of a Deeply Conserved Eumetazoan SINE Domain Mol Biol Evol 2011 28 2727 2730 10.1093/molbev/msr115 21512106

[B12] Arkhipova IR Distribution and phylogeny of Penelope-like elements in eukaryotes Syst Biol 2006 55 875 885 10.1080/10635150601077683 17345670

[B13] Pritham EJ Putliwala T Feschotte C Mavericks, a novel class of giant transposable elements widespread in eukaryotes and related to DNA viruses Gene 2007 390 3 17 10.1016/j.gene.2006.08.008 17034960

[B14] Kapitonov VV Jurka J Self-synthesizing DNA transposons in eukaryotes Proc Natl Acad Sci USA 2006 103 4540 4545 10.1073/pnas.0600833103 16537396 PMC1450207

[B15] Jurka J Kapitonov VV Pavlicek A Klonowski P Kohany O Walichiewicz J Repbase Update, a database of eukaryotic repetitive elements Cytogenet Genome Res 2005 110 462 467 10.1159/000084979 16093699

[B16] Piednoël M Bonnivard E DIRS1-like retrotransposons are widely distributed among Decapoda and are particularly present in hydrothermal vent organisms BMC Evol Biol 2009 9 86 10.1186/1471-2148-9-86 19400949 PMC2685390

[B17] Smit A Hubley R Green P RepeatMasker Open-3.0 1996 http://www.repeatmasker.org

[B18] Lerat E Identifying repeats and transposable elements in sequenced genomes: how to find your way through the dense forest of programs Heredity 2010 104 520 533 10.1038/hdy.2009.165 19935826

[B19] Ellinghaus D Kurtz S Willhoeft U LTRharvest, an efficient and flexible software for de novo detection of LTR retrotransposons BMC Bioinformatics 2008 9 18 10.1186/1471-2105-9-18 18194517 PMC2253517

[B20] Bao Z Eddy SR Automated de novo identification of repeat sequence families in sequenced genomes Genome Res 2002 12 1269 1276 10.1101/gr.88502 12176934 PMC186642

[B21] Goodwin TJD Poulter RTM Lorenzen MD Beeman RW DIRS retroelements in arthropods: identification of the recently active TcDirs1 element in the red flour beetle Tribolium castaneum Mol Genet Genomics 2004 272 47 56 15221458 10.1007/s00438-004-1028-2

[B22] Chung S Zuker C Lodish HF A repetitive and apparently transposable DNA sequence in Dictyostelium discoideum associated with developmentally regulated RNAs Nucleic Acids Res 1983 11 4835 4852 10.1093/nar/11.14.4835 6308562 PMC326089

[B23] Cappello J Cohen SM Lodish HF Dictyostelium transposable element DIRS-1 preferentially inserts into DIRS-1 sequences Mol Cell Biol 1984 4 2207 2213 6095047 10.1128/mcb.4.10.2207-2213.1984 PMC369040

[B24] Colbourne JK Pfrender ME Gilbert D Thomas WK Tucker A Oakley TH Tokishita S Aerts A Arnold GJ Basu MK Bauer DJ Cáceres CE Carmel L Casola C Choi J Detter JC Dong Q Dusheyko S Eads BD Fröhlich T Geiler-Samerotte KA Gerlach D Hatcher P Jogdeo S Krijgsveld J Kriventseva EV Kültz D Laforsch C Lindquist E Lopez J Manak JR Muller J Pangilinan J Patwardhan RP Pitluck S Pritham EJ Rechtsteiner A Rho M Rogozin IB Sakarya O Salamov A Schaack S Shapiro H Shiga Y Skalitzky C Smith Z Souvorov A Sung W Tang Z Tsuchiya D Tu H Vos H Wang M Wolf YI Yamagata H Yamada T Ye Y Shaw JR Andrews J Crease TJ Tang H Lucas SM Robertson HM Bork P Koonin EV Zdobnov EM Grigoriev IV Lynch M Boore JL The ecoresponsive genome of Daphnia pulex Science 2011 331 555 561 10.1126/science.1197761 21292972 PMC3529199

[B25] Rho M Schaack S Gao X Kim S Lynch M Tang H LTR retroelements in the genome of Daphnia pulex BMC Genomics 2010 11 425 10.1186/1471-2164-11-425 20618961 PMC2996953

[B26] Altschul SF Gish W Miller W Myers EW Lipman DJ Basic local alignment search tool J Mol Biol 1990 215 403 410 2231712 10.1016/S0022-2836(05)80360-2

[B27] Bonasio R Zhang G Ye C Mutti NS Fang X Qin N Donahue G Yang P Li Q Li C Zhang P Huang Z Berger SL Reinberg D Wang J Liebig J Genomic comparison of the ants Camponotus floridanus and Harpegnathos saltator Science 2010 329 1068 1071 10.1126/science.1192428 20798317 PMC3772619

[B28] Wurm Y Wang J Riba-Grognuz O Corona M Nygaard S Hunt BG Ingram KK Falquet L Nipitwattanaphon M Gotzek D Dijkstra MB Oettler J Comtesse F Shih C Wu W Yang C Thomas J Beaudoing E Pradervand S Flegel V Cook ED Fabbretti R Stockinger H Long L Farmerie WG Oakey J Boomsma JJ Pamilo P Yi SV Heinze J Goodisman MAD Farinelli L Harshman K Hulo N Cerutti L Xenarios I Shoemaker D Keller L The genome of the fire ant Solenopsis invicta Proc Natl Acad Sci USA 2011 10.1073/pnas.1009690108 PMC3078418 21282665

[B29] Wurm Y Uva P Ricci F Wang J Jemielity S Iseli C Falquet L Keller L Fourmidable: a database for ant genomics BMC Genomics 2009 10 5 10.1186/1471-2164-10-5 19126223 PMC2639375

[B30] Smith CR Smith CD Robertson HM Helmkampf M Zimin A Yandell M Holt C Hu H Abouheif E Benton R Cash E Croset V Currie CR Elhaik E Elsik CG Favé M Fernandes V Gibson JD Graur D Gronenberg W Grubbs KJ Hagen DE Viniegra ASI Johnson BR Johnson RM Khila A Kim JW Mathis KA Munoz-Torres MC Murphy MC Mustard JA Nakamura R Niehuis O Nigam S Overson RP Placek JE Rajakumar R Reese JT Suen G Tao S Torres CW Tsutsui ND Viljakainen L Wolschin F Gadau J Draft genome of the red harvester ant Pogonomyrmex barbatus Proc Natl Acad Sci USA 2011 10.1073/pnas.1007901108 PMC3078412 21282651

[B31] Smith CD Zimin A Holt C Abouheif E Benton R Cash E Croset V Currie CR Elhaik E Elsik CG Fave M Fernandes V Gadau J Gibson JD Graur D Grubbs KJ Hagen DE Helmkampf M Holley J Hu H Viniegra ASI Johnson BR Johnson RM Khila A Kim JW Laird J Mathis KA Moeller JA Muñoz-Torres MC Murphy MC Nakamura R Nigam S Overson RP Placek JE Rajakumar R Reese JT Robertson HM Smith CR Suarez AV Suen G Suhr EL Tao S Torres CW van Wilgenburg E Viljakainen L Walden KKO Wild AL Yandell M Yorke JA Tsutsui ND Draft genome of the globally widespread and invasive Argentine ant (Linepithema humile) Proc Natl Acad Sci USA 2011 10.1073/pnas.1008617108 PMC3078359 21282631

[B32] Altschul SF Madden TL Schäffer AA Zhang J Zhang Z Miller W Lipman DJ Gapped BLAST and PSI-BLAST: a new generation of protein database search programs Nucleic Acids Res 1997 25 3389 3402 10.1093/nar/25.17.3389 9254694 PMC146917

[B33] Van Dongen S Graph Clustering by Flow Simulation 2000

[B34] De Palmenaer D Siguier P Mahillon J IS4 family goes genomic BMC Evol Biol 2008 8 18 10.1186/1471-2148-8-18 18215304 PMC2266710

[B35] Siguier P Gagnevin L Chandler M The new IS1595 family, its relation to IS1 and the frontier between insertion sequences and transposons Res Microbiol 2009 160 232 241 10.1016/j.resmic.2009.02.003 19286454

[B36] Edgar RC MUSCLE: multiple sequence alignment with high accuracy and high throughput Nucleic Acids Res 2004 32 1792 1797 10.1093/nar/gkh340 15034147 PMC390337

[B37] Castresana J Selection of conserved blocks from multiple alignments for their use in phylogenetic analysis Mol Biol Evol 2000 17 540 552 10742046 10.1093/oxfordjournals.molbev.a026334

[B38] Tamura K Peterson D Peterson N Stecher G Nei M Kumar S MEGA5: Molecular Evolutionary Genetics Analysis Using Maximum Likelihood, Evolutionary Distance, and Maximum Parsimony Methods Mol Biol Evol 2011 28 2731 2739 10.1093/molbev/msr121 21546353 PMC3203626

[B39] Jones DT Taylor WR Thornton JM The rapid generation of mutation data matrices from protein sequences Comput Appl Biosci 1992 8 275 282 1633570 10.1093/bioinformatics/8.3.275

[B40] Milne I Wright F Rowe G Marshall DF Husmeier D McGuire G TOPALi: software for automatic identification of recombinant sequences within DNA multiple alignments Bioinformatics 2004 20 1806 1807 10.1093/bioinformatics/bth155 14988107

[B41] Felsenstein J Confidence limits on phylogenies: an approach using the bootstrap Evolution 1985 39 783 791 10.2307/2408678 28561359

[B42] Hibbett DS Binder M Bischoff JF Blackwell M Cannon PF Eriksson OE Huhndorf S James T Kirk PM Lücking R Thorsten Lumbsch H Lutzoni F Matheny PB McLaughlin DJ Powell MJ Redhead S Schoch CL Spatafora JW Stalpers JA Vilgalys R Aime MC Aptroot A Bauer R Begerow D Benny GL Castlebury LA Crous PW Dai Y Gams W Geiser DM Griffith GW Gueidan C Hawksworth DL Hestmark G Hosaka K Humber RA Hyde KD Ironside JE Kõljalg U Kurtzman CP Larsson K Lichtwardt R Longcore J Miadlikowska J Miller A Moncalvo J Mozley-Standridge S Oberwinkler F Parmasto E Reeb V Rogers JD Roux C Ryvarden L Sampaio JP Schüssler A Sugiyama J Thorn RG Tibell L Untereiner WA Walker C Wang Z Weir A Weiss M White MM Winka K Yao Y Zhang N A higher-level phylogenetic classification of the Fungi Mycol Res 2007 111 509 547 10.1016/j.mycres.2007.03.004 17572334

[B43] Keeling PJ Burger G Durnford DG Lang BF Lee RW Pearlman RE Roger AJ Gray MW The tree of eukaryotes Trends Ecol Evol (Amst.) 2005 20 670 676 10.1016/j.tree.2005.09.005 16701456

[B44] Dunn CW Hejnol A Matus DQ Pang K Browne WE Smith SA Seaver E Rouse GW Obst M Edgecombe GD Sørensen MV Haddock SHD Schmidt-Rhaesa A Okusu A Kristensen RM Wheeler WC Martindale MQ Giribet G Broad phylogenomic sampling improves resolution of the animal tree of life Nature 2008 452 745 749 10.1038/nature06614 18322464

[B45] Philippe H Derelle R Lopez P Pick K Borchiellini C Boury-Esnault N Vacelet J Renard E Houliston E Quéinnec E Da Silva C Wincker P Le Guyader H Leys S Jackson DJ Schreiber F Erpenbeck D Morgenstern B Wörheide G Manuel M Phylogenomics revives traditional views on deep animal relationships Curr Biol 2009 19 706 712 10.1016/j.cub.2009.02.052 19345102

[B46] Aparicio S Chapman J Stupka E Putnam N Chia J Dehal P Christoffels A Rash S Hoon S Smit A Gelpke MDS Roach J Oh T Ho IY Wong M Detter C Verhoef F Predki P Tay A Lucas S Richardson P Smith SF Clark MS Edwards YJK Doggett N Zharkikh A Tavtigian SV Pruss D Barnstead M Evans C Baden H Powell J Glusman G Rowen L Hood L Tan YH Elgar G Hawkins T Venkatesh B Rokhsar D Brenner S Whole-genome shotgun assembly and analysis of the genome of Fugu rubripes Science 2002 297 1301 1310 10.1126/science.1072104 12142439

[B47] Zuker C Cappello J Chisholm RL Lodish HF A repetitive Dictyostelium gene family that is induced during differentiation and by heat shock Cell 1983 34 997 1005 10.1016/0092-8674(83)90557-3 6194894

[B48] Putnam NH Srivastava M Hellsten U Dirks B Chapman J Salamov A Terry A Shapiro H Lindquist E Kapitonov VV Jurka J Genikhovich G Grigoriev IV Lucas SM Steele RE Finnerty JR Technau U Martindale MQ Rokhsar DS Sea anemone genome reveals ancestral eumetazoan gene repertoire and genomic organization Science 2007 317 86 94 10.1126/science.1139158 17615350

[B49] Ruiz-Pérez VL Murillo FJ Torres-Martínez S Prt1, an unusual retrotransposon-like sequence in the fungus Phycomyces blakesleeanus Mol Gen Genet 1996 253 324 333 9003319 10.1007/pl00008599

[B50] Volff J Lehrach H Reinhardt R Chourrout D Retroelement dynamics and a novel type of chordate retrovirus-like element in the miniature genome of the tunicate Oikopleura dioica Mol Biol Evol 2004 21 2022 2033 10.1093/molbev/msh207 15254255

[B51] Eichinger L Pachebat JA Glöckner G Rajandream M Sucgang R Berriman M Song J Olsen R Szafranski K Xu Q Tunggal B Kummerfeld S Madera M Konfortov BA Rivero F Bankier AT Lehmann R Hamlin N Davies R Gaudet P Fey P Pilcher K Chen G Saunders D Sodergren E Davis P Kerhornou A Nie X Hall N Anjard C Hemphill L Bason N Farbrother P Desany B Just E Morio T Rost R Churcher C Cooper J Haydock S van Driessche N Cronin A Goodhead I Muzny D Mourier T Pain A Lu M Harper D Lindsay R Hauser H James K Quiles M Madan Babu M Saito T Buchrieser C Wardroper A Felder M Thangavelu M Johnson D Knights A Loulseged H Mungall K Oliver K Price C Quail MA Urushihara H Hernandez J Rabbinowitsch E Steffen D Sanders M Ma J Kohara Y Sharp S Simmonds M Spiegler S Tivey A Sugano S White B Walker D Woodward J Winckler T Tanaka Y Shaulsky G Schleicher M Weinstock G Rosenthal A Cox EC Chisholm RL Gibbs R Loomis WF Platzer M Kay RR Williams J Dear PH Noegel AA Barrell B Kuspa A The genome of the social amoeba Dictyostelium discoideum Nature 2005 435 43 57 10.1038/nature03481 15875012 PMC1352341

[B52] Hellsten U Harland RM Gilchrist MJ Hendrix D Jurka J Kapitonov V Ovcharenko I Putnam NH Shu S Taher L Blitz IL Blumberg B Dichmann DS Dubchak I Amaya E Detter JC Fletcher R Gerhard DS Goodstein D Graves T Grigoriev IV Grimwood J Kawashima T Lindquist E Lucas SM Mead PE Mitros T Ogino H Ohta Y Poliakov AV Pollet N Robert J Salamov A Sater AK Schmutz J Terry A Vize PD Warren WC Wells D Wills A Wilson RK Zimmerman LB Zorn AM Grainger R Grammer T Khokha MK Richardson PM Rokhsar DS The genome of the Western clawed frog Xenopus tropicalis Science 2010 328 633 636 10.1126/science.1183670 20431018 PMC2994648

[B53] Putnam NH Butts T Ferrier DEK Furlong RF Hellsten U Kawashima T Robinson-Rechavi M Shoguchi E Terry A Yu J Benito-Gutiérrez EL Dubchak I Garcia-Fernàndez J Gibson-Brown JJ Grigoriev IV Horton AC de Jong PJ Jurka J Kapitonov VV Kohara Y Kuroki Y Lindquist E Lucas S Osoegawa K Pennacchio LA Salamov AA Satou Y Sauka-Spengler T Schmutz J Shin-I T Toyoda A Bronner-Fraser M Fujiyama A Holland LZ Holland PWH Satoh N Rokhsar DS The amphioxus genome and the evolution of the chordate karyotype Nature 2008 453 1064 1071 10.1038/nature06967 18563158

[B54] Merchant SS Prochnik SE Vallon O Harris EH Karpowicz SJ Witman GB Terry A Salamov A Fritz-Laylin LK Maréchal-Drouard L Marshall WF Qu L Nelson DR Sanderfoot AA Spalding MH Kapitonov VV Ren Q Ferris P Lindquist E Shapiro H Lucas SM Grimwood J Schmutz J Cardol P Cerutti H Chanfreau G Chen C Cognat V Croft MT Dent R Dutcher S Fernández E Fukuzawa H González-Ballester D González-Halphen D Hallmann A Hanikenne M Hippler M Inwood W Jabbari K Kalanon M Kuras R Lefebvre PA Lemaire SD Lobanov AV Lohr M Manuell A Meier I Mets L Mittag M Mittelmeier T Moroney JV Moseley J Napoli C Nedelcu AM Niyogi K Novoselov SV Paulsen IT Pazour G Purton S Ral J Riaño-Pachón DM Riekhof W Rymarquis L Schroda M Stern D Umen J Willows R Wilson N Zimmer SL Allmer J Balk J Bisova K Chen C Elias M Gendler K Hauser C Lamb MR Ledford H Long JC Minagawa J Page MD Pan J Pootakham W Roje S Rose A Stahlberg E Terauchi AM Yang P Ball S Bowler C Dieckmann CL Gladyshev VN Green P Jorgensen R Mayfield S Mueller-Roeber B Rajamani S Sayre RT Brokstein P Dubchak I Goodstein D Hornick L Huang YW Jhaveri J Luo Y Martínez D Ngau WCA Otillar B Poliakov A Porter A Szajkowski L Werner G Zhou K Grigoriev IV Rokhsar DS Grossman AR The Chlamydomonas genome reveals the evolution of key animal and plant functions Science 2007 318 245 250 10.1126/science.1143609 17932292 PMC2875087

[B55] Prochnik SE Umen J Nedelcu AM Hallmann A Miller SM Nishii I Ferris P Kuo A Mitros T Fritz-Laylin LK Hellsten U Chapman J Simakov O Rensing SA Terry A Pangilinan J Kapitonov V Jurka J Salamov A Shapiro H Schmutz J Grimwood J Lindquist E Lucas S Grigoriev IV Schmitt R Kirk D Rokhsar DS Genomic analysis of organismal complexity in the multicellular green alga Volvox carteri Science 2010 329 223 226 10.1126/science.1188800 20616280 PMC2993248

[B56] Colbourne JK Pfrender ME Gilbert D Thomas WK Tucker A Oakley TH Tokishita S Aerts A Arnold GJ Basu MK Bauer DJ Cáceres CE Carmel L Casola C Choi J Detter JC Dong Q Dusheyko S Eads BD Fröhlich T Geiler-Samerotte KA Gerlach D Hatcher P Jogdeo S Krijgsveld J Kriventseva EV Kültz D Laforsch C Lindquist E Lopez J Manak JR Muller J Pangilinan J Patwardhan RP Pitluck S Pritham EJ Rechtsteiner A Rho M Rogozin IB Sakarya O Salamov A Schaack S Shapiro H Shiga Y Skalitzky C Smith Z Souvorov A Sung W Tang Z Tsuchiya D Tu H Vos H Wang M Wolf YI Yamagata H Yamada T Ye Y Shaw JR Andrews J Crease TJ Tang H Lucas SM Robertson HM Bork P Koonin EV Zdobnov EM Grigoriev IV Lynch M Boore JL The ecoresponsive genome of Daphnia pulex Science 2011 331 555 561 10.1126/science.1197761 21292972 PMC3529199

[B57] Fritz-Laylin LK Prochnik SE Ginger ML Dacks JB Carpenter ML Field MC Kuo A Paredez A Chapman J Pham J Shu S Neupane R Cipriano M Mancuso J Tu H Salamov A Lindquist E Shapiro H Lucas S Grigoriev IV Cande WZ Fulton C Rokhsar DS Dawson SC The genome of Naegleria gruberi illuminates early eukaryotic versatility Cell 2010 140 631 642 10.1016/j.cell.2010.01.032 20211133

[B58] Xia Q Zhou Z Lu C Cheng D Dai F Li B Zhao P Zha X Cheng T Chai C Pan G Xu J Liu C Lin Y Qian J Hou Y Wu Z Li G Pan M Li C Shen Y Lan X Yuan L Li T Xu H Yang G Wan Y Zhu Y Yu M Shen W Wu D Xiang Z Yu J Wang J Li R Shi J Li H Li G Su J Wang X Li G Zhang Z Wu Q Li J Zhang Q Wei N Xu J Sun H Dong L Liu D Zhao S Zhao X Meng Q Lan F Huang X Li Y Fang L Li C Li D Sun Y Zhang Z Yang Z Huang Y Xi Y Qi Q He D Huang H Zhang X Wang Z Li W Cao Y Yu Y Yu H Li J Ye J Chen H Zhou Y Liu B Wang J Ye J Ji H Li S Ni P Zhang J Zhang Y Zheng H Mao B Wang W Ye C Li S Wang J Wong GK Yang H A draft sequence for the genome of the domesticated silkworm (Bombyx mori) Science 2004 306 1937 1940 15591204 10.1126/science.1102210

[B59] Ma L Ibrahim AS Skory C Grabherr MG Burger G Butler M Elias M Idnurm A Lang BF Sone T Abe A Calvo SE Corrochano LM Engels R Fu J Hansberg W Kim J Kodira CD Koehrsen MJ Liu B Miranda-Saavedra D O'Leary S Ortiz-Castellanos L Poulter R Rodriguez-Romero J Ruiz-Herrera J Shen Y Zeng Q Galagan J Birren BW Cuomo CA Wickes BL Genomic analysis of the basal lineage fungus Rhizopus oryzae reveals a whole-genome duplication PLoS Genet 2009 5 e1000549 10.1371/journal.pgen.1000549 19578406 PMC2699053

