# Supplementary figures and images for: Eukaryote DIRS1-like retrotransposons: an overview
Source: BMC Genomics. 2011 Dec 20;12:621. doi: 10.1186/1471-2164-12-621 (PMC3266345; doi:10.1186/1471-2164-12-621)

**A**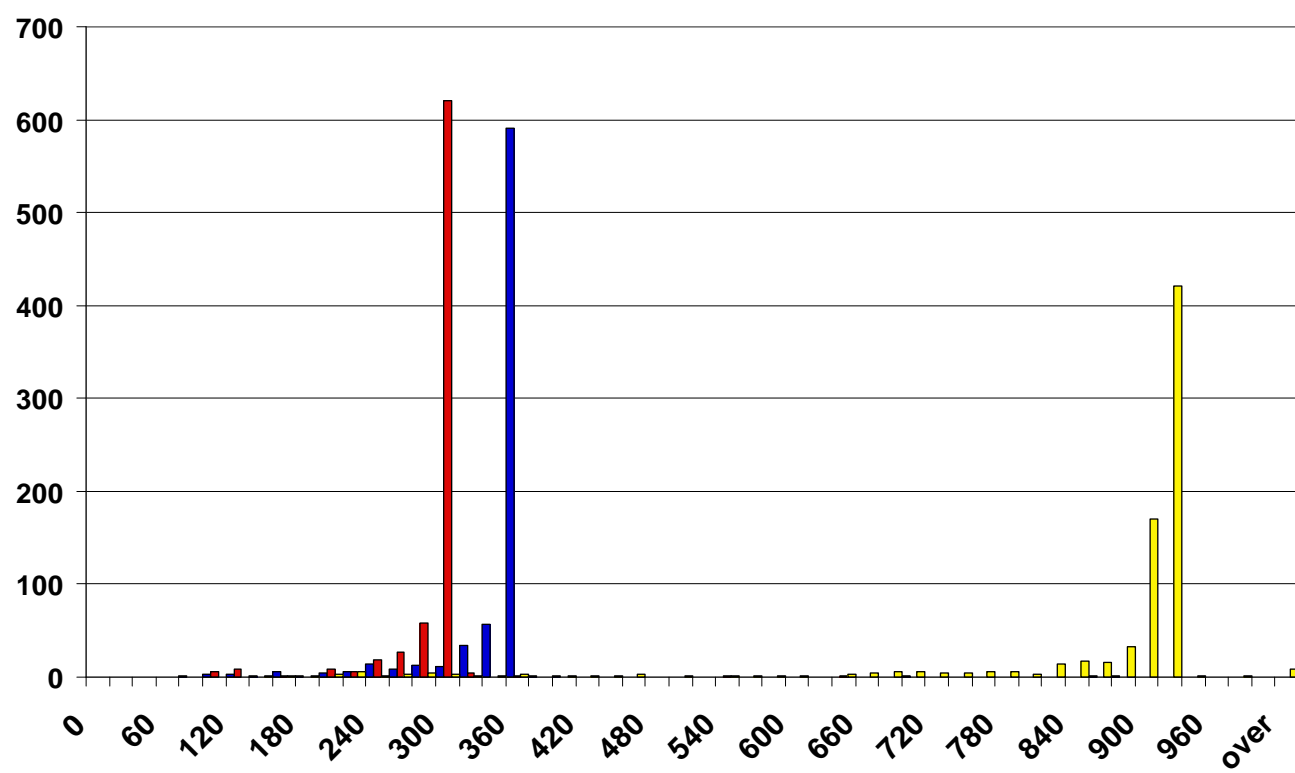**B**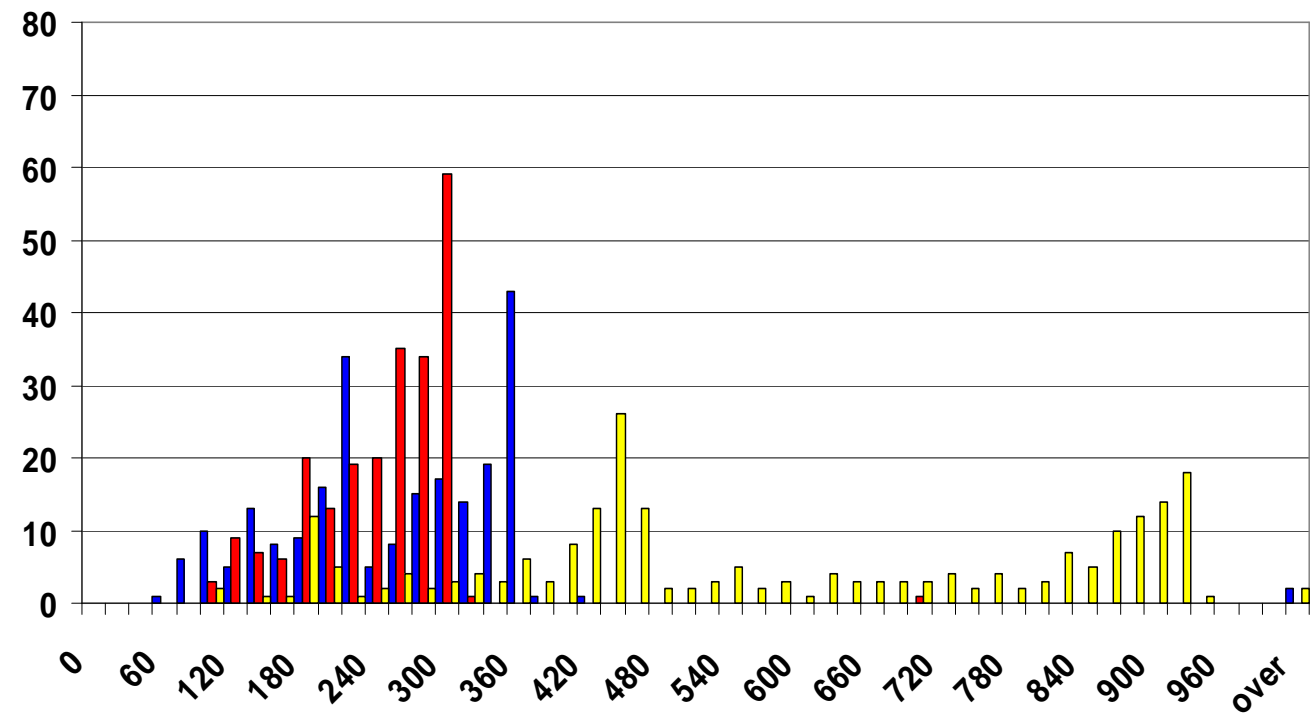

Supplement: Additional file 1 — Domain size distributions for the elements detected in A. carolinensis (A) and S. kowalevskii (B). The histogram represents the number of element domains detected (y-axis) as a function of their length (x-axis). The reverse transcriptase fragments are represented in blue, the methyltransferase fragments in red, and the tyrosine recombinase fragments in yellow. [file 1471-2164-12-621-S1.PDF]

## Slide 1
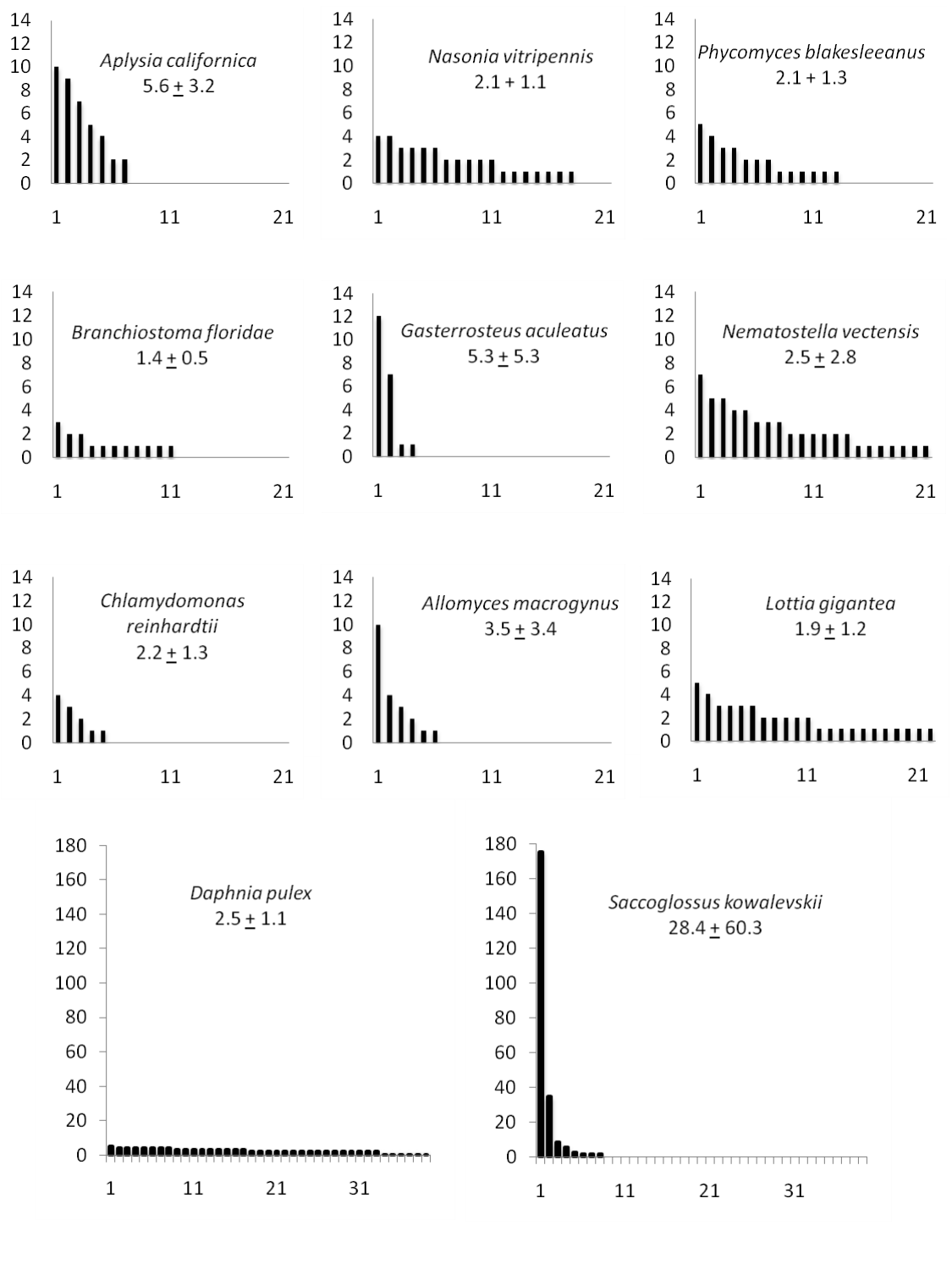

Supplement: Additional file 2 — Distribution of family size. Families are arranged along a gradient of decreasing size. For each species, mean family size and standard deviation are given. X-axis: family rank, Y-axis: number of elements in the family. [file 1471-2164-12-621-S2.PPT]
