# Supplementary material for: Eukaryote DIRS1-like retrotransposons: an overview
Source: BMC Genomics. 2011 Dec 20;12:621. doi: 10.1186/1471-2164-12-621 (PMC3266345; doi:10.1186/1471-2164-12-621)
Supplement: Additional file 4 — ReDoSt pipeline and alignment profiles used in this study. [file 1471-2164-12-621-S4.ZIP › Additional File 5/index.html]

ReDoSt


### ReDoSt

ReDoSt (Retrotransposon Domain Structure) is a computational tool dedicated to the identification of DIRS1-like retrotransposons in genomes  
The automatic element detection is based on both similarity searches using alignment profiles and the usual struture of DIRS1-like retrotransposons  
ReDoSt is publicaly available and can download from source files  
Before installation and use, please read Help  
If you are using the software, please cite...Piednoël M., Gonçalves I.R., Higuet D. and Bonnivard E. *Eukaryote DIRS1-like retrotransposons : an overview.*. 2011, submitted.  
If you have any comment, suggestion or bug report, or if you want to be kept informed about possible updates,  
please contact  M. Piednoël  
