# Supplementary material for: Eukaryote DIRS1-like retrotransposons: an overview
Source: BMC Genomics. 2011 Dec 20;12:621. doi: 10.1186/1471-2164-12-621 (PMC3266345; doi:10.1186/1471-2164-12-621)
Supplement: Additional file 5 — List of all species tested. For each species, the acronym used during the study and the data source website are indicated. [file 1471-2164-12-621-S5.PDF]

Acronym	Species	Data Source
Aa	*Ascosphaera apis*	baylor college of medecine
Aae	*Aedes aegypti*	broad institute
Aan	*Aureococcus anophagefferens*	jgi
Ab	*Alternaria brassicicola*	wustl
Ac	*Anolis carolinensis*	broadinstiute
Aca	*Aplysia californica*	broad institute
Acas	*Acanthamoeba castellanii*	hgsc
Acl	*Aspergillus clavatus*	broad institute
Ad	*Ajellomyces dermatitidis ER-3*	ncbi
Af	*Aspergillus fumigatus*	broad institute
Afl	*Aspergillus flavus*	broad institute
Ag	*Anopheles gambiae M*	wustl
Al	*Antonospora locustae*	antonospora locustae db
Aly	*Arabidopsis lyrata*	jgi
Am	*Allomyces macrogynus*	broad institute
Ame	*Apis mellifera*	hgsc
An	*Aspergillus nidulans*	broad institute
Ani	*Aspergillus niger*	broad institute
Ao	*Aspergillus oryzae*	broad institute
Ap	*Acyrthosiphon pisum*	hgsc
As	*Ascaris suum*	nematode.net
At	*Aspergillus terreus*	broad institute
Ath	*Arabidopsis thaliana (v9)*	http://www.arabidopsis.org/
Bb	*Babesia bovis*	ncbi
Bbi	*Babesia bigemina*	sanger
Bc	*Botrytis cinerea*	broad institute
Bd	*Batrachochytrium dendrobatidis*	broad institute
Bde	*Blastomyces dermatitidis SLH14081*	broad institute
Bdi	*Brachypodium distachyon*	brachybase
Bf	*Branchiostoma floridae*	jgi
Bm	*Bombyx mori*	silkworm database
Bt	*Bos taurus*	hgsc
Ca	*Candida albicans WO1*	broad institute
Casp	*Capitella sp. I ESC-2004*	jgi
Cb	*Caenorhabditis briggsae*	http://genome.ucsc.edu/cgi-bin/
Cbr	*Caenorhabditis brenneri*	wustl
Cc	*Coprinosus cinereus*	broad institute
Cd	*Candida dubliniensis*	sanger
Ce	*Caenorhabditis elegans*	ncbi
Cf	*Canis familiaris 09/09/05*	ncbi
Cg	*Candida guilliermondii*	broad institute
Cgl	*Chaetomium globosum*	broad institute
Cgla	*Candida glabrata*	genolevures
Cgr	*Colletotrichum graminicola M1.001*	broad institute
Ch	*Choloepus hoffmanni*	broad institute
Che	*Cochliobolus heterostrophus*	jgi
Cho	*Cryptosporidium hominis*	cryptoDB
Ci	*Coccidioides immitis RS*	broad institute
Cin	*Ciona intestinalis*	jgi
Cj	*Callithrix jacchus*	wustl
Cja	*Caenorhabditis japonica*	wustl
Cl	*Candida lusitaniae*	broad institute
Cm	*Cyanidioschyzon merolae*	http://merolae.biol.s.u-tokyo.ac.jp/download/
Cmi	*Callorhinchus milii*	esharkgenome.imcb
Cmu	*Cryptosporidium muris*	cryptoDB

Cn	*Cryptococcus neoformans*	broad institute
Co	*Capsaspora owczarzaki*	broad institute
Cos	*Coccomyxa sp.*	jgi
Cp	*Cavia porcellus*	UCSC cavPor3
Cpa	*Candida parapsilosis*	broad institute
Cpap	*Carica papaya*	ftp://asgpb.mhpcc.hawaii.edu/papaya/assembly/
Cpar	*Cryptosporidium parvum*	cryptoDB
Cpi	*Culex pipiens*	broad institute
Cpo	*Coccidioides posadasii Silveira*	broad institute
Cr	*Chlamydomonas reinhardtii*	jgi
Cre	*Caenorhabditis remanei*	wustl
Cs	*Ciona savignyi*	ensembl v2.0.56
Csa	*Cucumis sativus*	Cucumber genome database
Csp	*Chlorella sp. NC64A*	jgi
Ct	*Candida tropicalis*	broad institute
Cv	*Chlorella vulgaris*	jgi
Da	*Drosophila ananassae*	flybase
Dd	*Dictyostelium discoideum*	DictyBase
De	*Drosophila erecta*	flybase
Dg	*Drosophila grimshawi*	flybase
Dh	*Debaryomyces hansenii*	broad institute
Dm	*Drosophila melanogaster*	flybase
Dmo	*Drosophila mojavensis*	flybase
Dn	*Dasypus novemicinctus*	broad institute
Do	*Dipodomys ordii*	broad institute
Dp	*Dictyostelium purpureum*	jgi
Dpe	*Drosophila persimilis*	flybase
Dps	*Drosophila pseudoobscura*	flybase
Dpu	*Daphnia pulex*	jgi
Dr	*Danio rerio*	ensembl
Ds	*Drosophila sechelia*	flybase
Dsi	*Drosophila simulans*	flybase
Dv	*Drosophila virilis*	flybase
Dw	*Drosophila willistoni*	flybase
Dy	*Drosophila yakuba*	flybase
Ec	*Equus caballus*	broad institute
Ecu	*Encephalitozoon cuniculi*	ncbi
Ee	*Erinaceus europeaus*	broad institute
Eg	*Eremothecium gossypii*	jgi
Eh	*Emiliana huxleyi*	jgi
Ehi	*Entamoeba histolytica*	sanger
Et	*Echinops telfari*	broad institute
Ete	*Eimeria tenella*	sanger
Fc	*Felis catus*	broad institute
Fcy	*Fragilariopsis cylindrus*	jgi
Fg	*Fusarium graminearum*	broad institute
Fo	*Fusarium oxysporum*	broad institute
Fv	*Fusarium verticillioides*	broad institute
Ga	*Gasterosteus aculeatus*	broad institute
Gg	*Gallus gallus*	wustl
Ggo	*Gorilla gorilla*	ensembl
Gi	*Giardia intestinalis*	ncbi
Gm	*Glycine max*	plantGDB
Gp	*Globodera pallida*	sanger
Gt	*Guillardia theta*	ncbi
Ha	*Heterobasidion annosum*	jgi

Han	*Hemiselmis andersenii*	ncbi
Hb	*Heterorhabditis bacteriophora*	wustl
Hc	*Histoplasma capsulatum NAm1*	broad institute
Hp	*Hyaloperonospora parasitica*	wustl
Hr	*Helobdella robusta*	jgi
Hs	*Homo sapiens*	ensembl
Is	*Ixodes scapularis*	vectorbase
Kl	*Kluyveromyces lactis*	genolevures
Kt	*Kluyveromyces thermotolerans*	genolevures
La	*Loxodonta africana*	broad institute
Lb	*Laccaria bicolor*	jgi
Lbr	*Leishmania braziliensis*	sanger
Le	*Lodderomyces elongisporus*	broad institute
Lg	*Lottia gigantea*	jgi
Li	*Leishmania infantum*	sanger
Lj	*Lotus japonicus*	kazusa
Lm	*Leishmania major*	sanger
Mb	*Monosiga brevicollis*	jgi
Mc	*Microsporum canis*	broad institute
Mci	*Mucor circinelloides*	jgi
Md	*Mayetiola destructor*	hgsc
Mdo	*Monodelphis domestica*	ncbi
Me	*Macropus eugenii*	hgsc
Mes	*Manihot esculenta*	jgi
Mf	*Mycosphaerella fijiensis*	jgi
Mg	*Microsporum gypseum*	broad institute
Mgl	*Malassezia globosa*	ncbi
Mgr	*Magnaporthe grisae 70-15*	broad institute
Mgra	*Mycosphaerella graminicola*	jgi
Mgu	*Mimulus guttatus*	jgi
Mh	*Meloidogyne hapla*	pngg
Mi	*Meloidogyne incognita*	wormbase
Ml	*Myotis lucifugus*	broad institute
Mla	*Melampsora laricis-populina*	jgi
Mm	*Microcebus murinus*	broad institute
Mmu	*Macaca mulatta*	hgsc
Mmus	*Mus musculus*	ncbi
Mp	*Micromonas pusilla CCMP1545*	jgi
Mt	*Medicago truncatula*	jcvi
Nc	*Neurospora crassa OR74A*	broad institute
Nca	*Neospora caninum*	sanger
Nd	*Neurospora discreta*	jgi
Nf	*Neosartorya fischeri*	broad institute
Ng	*Naegleria gruberi*	jgi
Nh	*Nectria haematococca*	jgi
Nt	*Neurospora tetrasperma*	jgi
Nv	*Nasonia vitripennis*	hgsc
Nve	*Nematostella vectensis*	jgi
Oa	*Ornithorhynchus anatinus*	wustl
Oc	*Oryctolagus cuniculus*	broad institute
Od	*Oikopleura dioica*	genoscope
Og	*Otolemur gametti*	broad institute
Oi	*Oryza indica*	gramene
Ol	*Ostreococcus 'lucimarinus'*	jgi
Ola	*Oryzias latipes*	UTGBMedaka
Op	*Ochotona princeps*	broad institute

Os	*Oryza sativa Japonica group*	http://rgp.dna.affrc.go.jp/
Osp	*Ostreococcus sp.*	jgi
Ot	*Ostreococcus tauri*	jgi
Pa	*Pongo abelii*	wustl
Pan	*Podospora anserina*	http://podospora.igmors.u-psud.fr/
Pb	*Paracoccitioides brasiliensis Pb01*	broad institute
Pbe	*Plasmodium berghei*	sanger
Pbl	*Phycomyces blakesleeanus*	jgi
Pc	*Procavia capensis*	broad institute
Pca	*Phytophtora capsici*	jgi
Pch	*Phanerochaete chrysosporium*	jgi
Pcha	*Plasmodium chabaudi*	sanger
Pd	*Phoenix dactylifera*	http://qatar-weill.cornell.edu/
Pf	*Plasmodium falciparum 3D7*	sanger
Pg	*Puccinia graminis tritici*	broad institute
Ph	*Papio hamadryas*	hgsc
Phu	*Pediculus humanus corporis*	vectorbase
Pk	*Plasmodium knowlesi*	sanger
Pm	*Penicillium marneffei*	ncbi
Pma	*Petromyzon marinus*	wustl
Po	*Pleurotus ostreatus*	jgi
Pp	*Pichia pastoris*	ncbi
Ppa	*Physcomitrella patens*	jgi
Ppac	*Pristionchus pacificus*	wustl
Ppl	*Postia placenta*	jgi
Ppo	*Physarium polycephalum*	Wustl
Pr	*Phytophtora ramorum*	jgi
Ps	*Pichia stipitis*	jgi
Pso	*Phytophtora sojae*	jgi
Psp	*Proterospongia sp.*	broad institute
Pt	*Pyrenophora tritici-repentis*	broad institute
Ptr	*Populus trichocarpa*	jgi
Ptri	*Phaeodactylum tricornutum*	jgi
Ptrit	*Puccinia triticina*	broad institute
Ptro	*Pan troglodytes*	wustl
Pu	*Pythium ultimum*	pythium genome database
Pv	*Pteropus vampyrus*	broad institute
Pvi	*Plasmodium vivax*	sanger
Rc	*Ricinus communis*	jcvi
Rn	*Rattus norvegicus*	hgsc
Ro	*Rhizopus oryzae RA 99-880*	broad institute
Rp	*Rhodnius prolixus*	wustl
Sa	*Sorex araneus*	broad institute
Sb	*Saccharomyces bayanus*	broad institute
Sbi	*Sorghum bicolor*	jgi
Sc	*Saccharomyces cerevisae*	SGD
Sco	*Schizophyllum commune*	jgi
Scr	*Schizosaccharomyces cryophobus OY26*	broad institute
Sj	*Schizosaccharomyces japonicus yFS275*	broad institute
Sja	*Schistosoma japonicum*	chgc
Sk	*Saccharomyces kluyveri*	Washihngton University School of Medecine
Sko	*Saccoglossus kowalevskii*	hgsc
Sl	*Serpula lacrymans*	jgi
Sly	*Solanium lycopersicum*	solgenomics
Sm	*Saccharomyces mikatae*	broad institute
Sma	*Schistosoma mansoni*	sanger

Sme	*Schmidtea mediterranea*	wustl
Smo	*Selaginella moellendorfii*	jgi
Sn	*Stagonospora nodorum*	broad institute
So	*Schizosaccharomyces octosporus yFS286*	broad institute
Sp	*Schizosaccharomyces pombe 9762h-*	broad institute
Spa	*Saccharomyces paradoxus*	broad institute
Sph	*Solanium phureja*	potatogenomics
Spu	*Spizellomyces punctatus*	broad institute
Spur	*Strongylocentrotus purpuratus*	hgsc
Sr	*Sporobolomyces roseus*	jgi
Ss	*Sclerotinia sclerotiorum*	broad institute
Ssc	*Sus scrofa*	ncbi
St	*Spermophilis tridecemlineatus*	broad institute
Sth	*Sporotrichum thermophile*	jgi
Sv	*Spironucleus vortens*	jgi
Ta	*Trichoderma atroviride*	jgi
Tad	*Trichoplax adhaerens*	jgi
Tan	*Theileria annulata*	sanger
Tb	*Tupaia belangeri*	broad institute
Tbr	*Trypanosoma brucei*	sanger
Tc2	*Tribolium castaneum v3*	beetlebase
Tcr	*Trypanosoma cruzi non esmeraldo*	TriTrypDB
Te	*Trichophyton equinum*	broad institute
Tg	*Taeniopygia guttata*	wustl
Tgo	*Toxoplasma gondii*	toxoDB
Tm	*Tremella mesenterica Fries*	jgi
Tn	*Tetraodon nigroviridis*	genoscope
Tp	*Thalassiosira pseudonana*	jgi
Tpa	*Theileria parva*	ncbi
Tr	*Takifugu rubripes*	joint genome institute
Tre	*Trichoderma reesei*	jgi
Tru	*Trichophyton rubrum*	broad institute
Ts	*Tarsier syrichta*	broad institute
Tsp	*Trichinella spiralis*	wustl
Tst	*Talaromyces stipitatus*	ncbi
Tt	*Tursiops truncatus*	broad institute
Tte	*Thielavia terrestris*	jgi
Tto	*Trichophyton tonsurans*	broad institute
Tv	*Trichoderma virens*	jgi
Tva	*Trichomonas vaginalis G3*	trichDB
Um	*Ustilago maydis*	broad institute
Ur	*Uncinicarpus reesii*	broad institute
Va	*Verticillium albo-atrum VaMs.102*	broad institute
Vc	*Volvox carteri*	jgi
Vd	*Verticillium dahliae VdLs.17*	broad institute
Vp	*Vicunia pacos*	broad institute
Vpo	*Vanderwaltozyma polyspora*	ncbi
Vv	*Vitis vinifera*	jgi
Xt	*Xenopus tropicalis*	jgi
Yl	*Yarrowia lipolytica*	genolevures
Zm	*Zea mais*	plantGDP
Zr	*Zygosaccharomyces rouxii*	genolevures
